# Rationally Engineered Heterometallic Metalladithiolene Coordination Nanosheets with Defined Atomic Arrangements

**DOI:** 10.1002/smll.202503227

**Published:** 2025-05-05

**Authors:** Miyu Ito, Naoya Fukui, Kenji Takada, Ziheng Yu, Hiroaki Maeda, Katsuya Mizuno, Hiroshi Nishihara

**Affiliations:** ^1^ Graduate School of Science and Technology Tokyo University of Science 2641 Yamazaki Noda Chiba 278‐8510 Japan; ^2^ Research Institute for Science and Technology Tokyo University of Science 2641 Yamazaki Noda Chiba 278‐8510 Japan

**Keywords:** colloid, coordination nanosheet, electrical conductivity, electrocatalyst, heterometal

## Abstract

Coordination nanosheets are 2D polymers formed by coordination bonds between metal ions and planar organic molecules. They offer high molecular design freedom and unique electronic and chemical properties, making them suitable for various usage. In the two‐phase interfacial reaction of metal ions with benzenehexathiol (BHT), highly conductive coordination nanosheets are generated. For nickel ions, porous NiDT (=Ni_3_BHT_2_) and nonporous NiBHT (=Ni_3_BHT) structures are obtained. However, a rational selective synthesis method for these structures is not well‐developed. In this study, selective synthesis of NiDT and NiBHT colloidal solutions is achieved by adjusting the BHT to metal ion ratio in a single phase. These colloidal solutions serve as hydrogen evolution catalysts by coating them on an electrode and NiDT is the better catalyst than NiBHT. CuBHT and ZnBHT colloidal solutions are also successfully prepared. Furthermore, nonporous NiCu_2_BHT and a new material, NiZn_2_BHT are synthesized by filling the NiDT pores with another metal and BHT. These approaches lead to the production of coordination nanosheet inks and the synthesis of heterometallic coordination nanosheets with controlled molecular structures. The introduction of the second metal significantly changes the electrical conductivity. Furthermore, a method is discovered to convert NiBHT to NiCu_2_BHT using a transmetallation reaction.

## Graphical abstract

Selective synthesis of NiDT and NiBHT colloidal solutions is achieved by adjusting the BHT to metal ion ratio in a single phase. Furthermore, nonporous NiCu_2_BHT and a new material, NiZn_2_BHT are synthesized, by filling the NiDT pores with another metal and BHT. A method is discovered to convert NiBHT to NiCu_2_BHT using a transmetallation reaction.

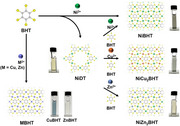

## 1 Introduction

Coordination nanosheets are 2D complex polymers formed by coordination bonds between metal ions and planar π‐conjugated organic molecules.^[^ ^1^, ^2^, ^3^, ^4^, ^5^, ^6^, ^7^ ^]^ There is a high degree of freedom in the structural design by the selection of metal ions and the molecular design of ligands, and the diverse combinations of these can produce unique physical and chemical properties. Because of the properties such as electronic, magnetic, optical, and redox properties and catalytic activity derived from the metal complex moiety, a wide range of applications such as energy storage, electronic devices, electrode materials, and catalysts are expected.^[^ ^8^, ^9^, ^10^, ^11^, ^12^, ^13^, ^14^, ^15^, ^16^, ^17^, ^18^, ^19^, ^20^, ^21^, ^22^, ^23^, ^24^, ^25^ ^]^ In 2013, we reported the synthesis of filmy multilayer and single‐layer porous nickelladithiolene nanosheets, NiDT (=Ni_3_BHT_2_), using the complexation reaction of benzenehexathiol (BHT) with Ni^2+^ ions at liquid‐liquid and gas‐liquid interfaces.^[^ ^26^ ^]^ This NiDT is synthesized in a mixed valence state of −3/4, but it showed chemically reversible redox behavior and revealed that the electrical conductivity was highly dependent on the oxidation state.^[^ ^27^ ^]^ The neutral multilayer NiDT obtained by chemically oxidizing as‐prepared NiDT has a metallic electronic structure with a conductivity of 160 S cm^−1^, and Liu et al. theoretically predicted that the single‐layer NiDT can be a 2D topological insulator.^[^ ^28^ ^]^

Subsequently, the synthesis of complex polymers by the reaction of various transition metal ions with BHT was reported.^[^ ^21^, ^24^, ^26^, ^29^, ^30^, ^31^, ^32^, ^33^, ^34^, ^35^ ^]^ In 2015, Huang et al. synthesized nonporous copper dithiolene nanosheets, CuBHT (=Cu_3_BHT), by reaction of Cu^2+^ ions with BHT.^[^ ^35^ ^]^ CuBHT exhibits a high conductivity of 2500 S cm^−1^ and superconductivity at 0.25K.^[^ ^36^, ^37^ ^]^ However, it is worth noting that in 2024, Pan et al. reported the results of the analysis of the single‐crystal structure of CuBHT, showing that it is not a true 2D structure, but a pseudo‐2D structure (3D network).^[^ ^38^ ^]^ Furthermore, Banda et al. and we reported that the reaction of Ni^2+^ ions with BHT also produces nonporous NiBHT (=Ni_3_BHT).^[^ ^33^, ^34^ ^]^ We reported that in the two‐phase interfacial reaction, the porous NiDT structure is formed in the presence of Na^+^ ions and the nonporous NiBHT structure is formed in the absence of Na^+^ ions.^[^ ^26^, ^34^ ^]^

**Figure 1:**
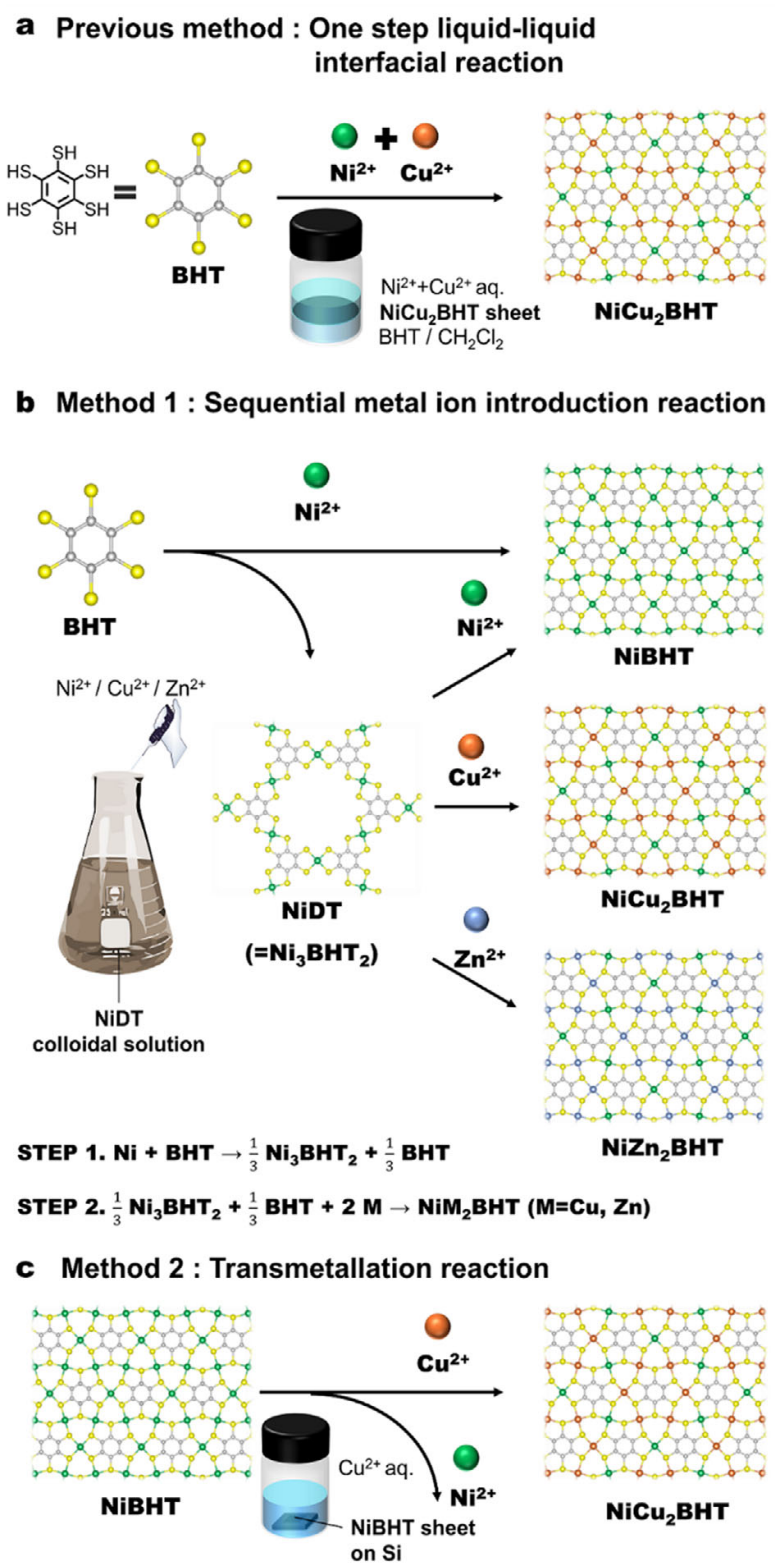
Schematic illustration of the preparation of heterometallic metalladithiolene nanosheets. a) Previous one‐step liquid‐liquid interfacial reaction. b) Sequential metal ion introduction reaction. c) Transmetallation reaction.

In recent years, in addition to single‐metal coordination nanosheets, heterometallic nanosheets formed by different metal ions and ligands have been synthesized.^[^ ^34^, ^39^, ^40^, ^41^, ^42^, ^43^, ^44^, ^45^, ^46^ ^]^ This is because the inclusion of multiple metals is expected to produce excellent chemical and physical properties. However, there have been few reports of heterometallic coordination nanosheets with regular arrangements because the nanosheets are mostly solid solutions in which the metal ions are randomly arranged. We have reported that a heterometallic coordination nanosheet, NiCu_2_BHT, with a structure in which Cu and BHT (Cu:BHT = 6:1) are introduced into the pores of NiDT can be synthesized by a complex formation reaction between a mixed aqueous solution of Ni^2+^ ions and Cu^2+^ ions and an organic solvent containing BHT in a two‐phase interfacial synthesis method (**Figure** **1**a).^[^ ^34^ ^]^ Compared to Ni_3_BHT and Cu_3_BHT, NiCu_2_BHT has high crystallinity and high electrical conductivity. This shows the superiority of the structure and physical properties of heterometallic coordination nanosheets with a defined structure, and the development of a more precise and versatile synthesis method is desired.

In this study, we achieved the synthesis of heterometallic coordination nanosheets using two new methods (Figure 1b,c). The first method uses a colloidal solution of coordination nanosheets prepared by a single‐phase reaction of metal ions and BHT. Until now, coordination nanosheets have been obtained as filmy products by two‐phase interface synthesis and as powdery products by single‐phase synthesis, but if the coordination nanosheets can be prepared as colloidal dispersion solutions, they can be used as inks to impart processability, such as coating on substrates, or as reaction solutions for subsequent reactions.^[^ ^34^, ^43^, ^47^, ^48^, ^49^, ^50^, ^51^, ^52^, ^53^, ^54^, ^55^, ^56^ ^]^ In addition, because the ratios of Ni and BHT in NiDT and NiBHT are different, the single‐phase reaction will realize a selective synthesis of the desired structure. Therefore, we first established the conditions for producing colloidal solutions of NiDT and NiBHT. Colloidal solutions of CuBHT and ZnBHT were also successfully synthesized by the same method as that used for NiBHT. Next, NiCu_2_BHT was successfully selectively synthesized by adding Cu^2+^ to the NiDT colloidal solution containing unreacted BHT (Figure 1b). This method suggests the possibility of synthesizing NiM_2_BHT, in which other metal ions, M^2+^, are inserted into NiDT. In fact, taking advantage of stepwise synthesis,^[^ ^57^ ^]^ we synthesized a new material, NiZn_2_BHT, by reacting a NiDT colloidal solution with Zn^2+^.

The second synthesis method for heterometallic coordination nanosheets uses the transmetallation reaction of NiBHT (Figure 1c). We found that a two‐step transmetallation reaction of ZnBHT led to the formation of a lateral heterojunction of tmFe/tmCu that exhibited rectifying properties.^[^ ^56^ ^]^ Herein we investigated the transmetallation reaction of NiBHT with Cu^2+^ and found that the two‐step transmetallation reaction proceeded kinetically to finally produce CuBHT, but the metastable product of the first step is NiCu_2_BHT.

In this paper, we report the synthesis, characterization and electrical conductivity of NiDT, NiBHT, NiCu_2_BHT, and NiZn_2_BHT. These results contribute significantly to the preparation of the colloidal solution of coordination nanosheets and their use in the synthesis of structure‐defined heterometallic coordination nanosheets. We also demonstrate that transmetallation reactions are useful for the synthesis of heterometallic coordination nanosheets.

## 2 Results and Discussion

### 2.1 Preparation of NiDT and MBHT Colloidal Solutions (M = Ni, Cu, Zn)

**Figure 2:**
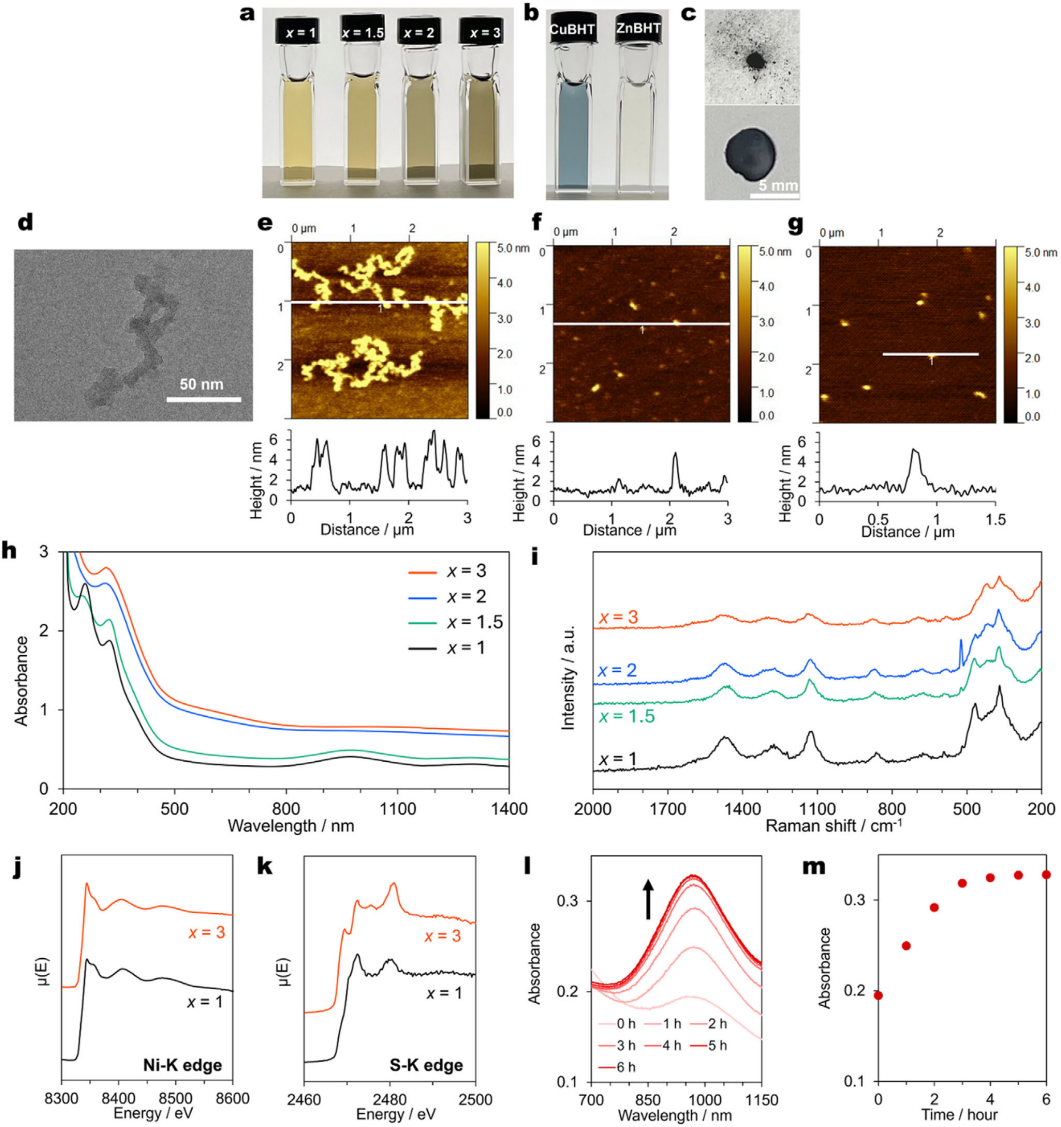
Synthesis and characterization of monometallic coordination nanosheet colloidal solutions. a) Photo of Ni*_x_*/BHT colloidal solutions. b) Photo of CuBHT and ZnBHT colloidal solutions. c) Photo of powder (top) and pellet (bottom) of Ni_1_/BHT. d) TEM image of Ni_1_/BHT. e–g) AFM topography images and height profiles of Ni_1_/BHT (e,f) and Ni_3_/BHT (g) at the corresponding white lines. h) UV–vis–NIR absorption spectra of Ni*_x_*/BHT colloidal solutions. i) Raman spectra of Ni*_x_*/BHTs. j) Ni K‐edge XANES spectra for Ni*_x_*/BHTs (*x* = 1, 3). k) S K‐edge XANES spectra of Ni*_x_*/BHTs (*x* = 1, 3). l) Time‐dependent vis–NIR absorption spectra of Ni_1_/BHT from 0 to 6 h. m) Dependency of absorbance at 975 nm on reaction time.

The selective synthesis of NiDT and NiBHT was investigated by varying the stoichiometric ratio of Ni^2+^ ions to BHT (*x* = [Ni^2+^]/[BHT]) in a single phase. To a 0.15 mm BHT/THF solution, the same volume (0.15 × *x*) mm (*x* = 1, 1.5, 2, 3) Ni(OAc)_2_/MeOH solution was quickly added and stirred. The reaction proceeded immediately and gave Ni*_x_*/BHT dispersions. The dispersion became darker as the stoichiometry of the Ni ions increased, being brown at *x* = 1 and 1.5 and dark green at *x* = 2 and 3 (**Figure** **2**a). No precipitation formed under any reaction conditions above, and homogeneous dispersions were maintained for at least 2 weeks (Figure S1, Supporting Information). Ni*_x_*/BHT can be collected as a black powder by concentration by evaporation of solvent, addition of hexane, filtration, and wash with MeOH and THF (Figure 2c).

The dynamic light scattering (DLS) measurements of Ni*_x_*/BHT showed mode diameters of 25 ± 5 nm at *x* = 1.5 and 43 ± 12 nm at *x* = 3 (Figure S2, Supporting Information). The mode diameter of Ni_3_/BHT did not show a significant time dependency after synthesis, indicating that the particle size was determined at the time of synthesis and the insignificant growth of the domain proceeded (Figure S2, Supporting Information). Atomic force microscope (AFM) and transmission electron microscope (TEM) images showed that Ni*_x_*/BHTs had irregular morphologies, including dendrite and round‐shaped structures (Figure 2d–g; Figure S3, Supporting Information). The observed structures differed from our prediction that Ni*_x_*/BHTs give flat plate geometry because of their 2D chemical structures. This result can be explained as follows: Regarding BHT as the nucleus of Ni*_x_*/BHT formation, BHT has coordination sites with Ni in three directions. When a BHT coordinates to the termini of Ni*_x_*/BHT, the remaining two coordination sites in the BHT are available for further growth of Ni*_x_*/BHT in two directions. Repeating the BHT coordination to the termini and the Ni*_x_*/BHT growth in two directions gives Ni*_x_*/BHTs with irregular dendritic structures, as observed in microscopic observations. This dendritic structure with many edges is thought to be the reason why it increased interaction with the solvent and decreased the attractive interaction between particles, leading to a colloidal solution without precipitation.^[^ ^58^, ^59^ ^]^ The thicknesses of Ni_1_/BHT and Ni_3_/BHT were ≈2–7 nm, corresponding to 6–20 layers (Figure 2e–g).^[^ ^26^, ^34^ ^]^

The UV–vis–NIR spectra of the Ni_1_/BHT and Ni_1.5_/BHT colloidal solutions recorded under an argon atmosphere exhibited an absorption peak at 970 nm derived from the π–π^*^ transition, corresponding to the peak of NiDT previously reported (Figure 2h).^[^ ^60^ ^]^ In contrast, Ni_3_/BHT colloidal solution gave an absorption peak of at ≈1070 nm which is consistent with the peak of NiBHT synthesized by liquid‐liquid interfacial synthesis (Figure 2h; Figure S4, Supporting Information). These results presume that the electronic structures of Ni*_x_*/BHTs depend on the stoichiometric ratio of Ni^2+^ ions and BHT in the synthesis.

When the Ni_1_/BHT dispersion solution was exposed to air, the absorption peak at 970 nm gradually increased in absorbance and shifted to ≈955 nm over 90 min (Figure S5, Supporting Information). The Raman spectrum of Ni_1_/BHT showed peaks ascribed to the Ni─S bond at 371 and 392 cm^−1^ under Ar. When the sample was exposed to air, a new peak appeared at ≈470 cm^−1^ (Figure S6, Supporting Information). X‐ray photoelectron spectra (XPS) of Ni_1_/BHT drop‐cast on a Si substrate under air and rinsed to remove unreacted BHT were consistent with NiDT in the neutral state prepared by chemical oxidation, suggesting that Ni_1_/BHT takes a neutral state after air exposure (Figure S7, Supporting Information).^[^ ^27^ ^]^ The experimental results described below are for Ni_1_/BHT exposed to air unless otherwise stated.

The Raman spectra of Ni*_x_*/BHTs showed peaks derived from C─S and C═C bonds stretching vibrations at 1100 and 1300–1600 cm^−1^, respectively, corresponding to the reported spectra of Ni_3_BHT (Figure 2i).^[^ ^34^ ^]^ All Ni*_x_*/BHTs also gave a peak at 370 cm^−1^ ascribed to Ni─S bond vibration. A peak at ≈470 cm^−1^ in Ni_1_/BHT gradually disappeared with increasing *x*, whereas a gradual appearance of a new peak at ≈415 cm^−1^ was observed. The observed Raman shift value for this newly appeared peak is consistent with that for NiBHT.^[^ ^34^ ^]^ This result can be explained by the difference in the coordination structures between NiDT and NiBHT; the S atom bonds to one Ni atom in the NiDT structure, whereas the S atom bonds to two Ni atoms in the NiBHT structure. Therefore, these results indicate that Ni_1_/BHT takes the NiDT structure, while Ni_3_/BHT does NiBHT. The NiDT and NiBHT structures coexist in Ni*_x_* BHTs (*x* = 1.5 and 2). The coordination environments of Ni and S in Ni*_x_*/BHTs (*x* = 1, 3) are investigated in detail using X‐ray absorption near edge structure (XANES). The Ni K‐edge spectra in XANES did not show significant differences between Ni_1_/BHT and Ni_3_/BHTs (Figure 2j). The peaks observed at 8333 and 8346 eV were consistent with those of NiBHT previously reported.^[^ ^23^, ^33^ ^]^ On the other hand, the S K‐edge spectra of Ni_1_/BHT and Ni_3_/BHT were different (Figure 2k). The spectrum of Ni_3_/BHT exhibited additional peaks at 2469 and 2475 eV compared to that of Ni_1_/BHT. This observation can be explained as follows. Both Ni atoms in the NiDT and NiBHT structures take the same coordination environment, coordinated by four S atoms. On the other hand, an S atom in the NiDT structure coordinates with one Ni atom, whereas the one in the NiBHT structure bridges two Ni atoms. XPS of Ni_3_/BHT was also consistent with that of NiBHT previously reported (Figure S7, Supporting Information).^[^ ^34^ ^]^ This series of results indicates that Ni_1_/BHT and Ni_3_/BHT take NiDT and NiBHT structures, respectively.

NiDT and NiBHT colloidal solutions can serve as inks for coating on a solid surface. We coated glassy carbon (GC) electrodes with NiDT and NiBHT to evaluate their electrochemical behavior. The modified electrodes showed thicker electric double‐layers than a bare GC electrode in the cyclic voltammograms (Figure S8a, Supporting Information). The double‐layer capacitances of NiDT and NiBHT were comparable, 8.4 and 10 µF, respectively (Figure S8b,c, Supporting Information). Meanwhile, NiDT exhibited a broad redox wave at ≈0.1 V versus Fc^+^/Fc while NiBHT did not show a redox wave at the corresponding potential. The redox potential of NiDT is accordance with that of NiDT obtained by a liquid‐liquid interfacial synthesis, suggesting the formation of porous structure.^[^ ^26^ ^]^ Hence, the structural difference between NiDT and NiBHT affects their redox properties. Our previous work showcased the electrocatalytic activity of NiDT film for the hydrogen evolution reaction (HER).^[^ ^24^ ^]^ Therefore, we also investigated the catalytic activities of NiDT and NiBHT for HER in a 0.5 m sulfuric acid aqueous solution (Figure S8a,c, Supporting Information). The NiDT‐coated GC showed the onset potential (*E*_onset_) at −395 mV and the overpotential reaching the current density of −10 mA cm^−2^ (*η_j_*_= 10_) of 467 mV (Figure S9a, Supporting Information). This result demonstrates the electric contact between NiDT and the GC electrode and the availability of NiDT colloid in water electrolysis. NiBHT also worked as HER electrocatalyst while an additional overpotential ≈40 mV was required to reach −10 mA cm^−2^. The difference in the coordination structures of NiDT and NiBHT may provide the different catalytic activities. The Tafel slopes of NiDT and NiBHT were 70, 86 mV dec^−1^, respectively (Figure S9b,c, Supporting Information). Judging from these values, the rate‐determining step of HER catalyzed by NiDT and NiBHT is supposed to be the Heyrovsky step, where coupling of a proton on the electrode and a proton in the solution takes place.^[^ ^61^ ^]^

CuBHT and ZnBHT colloidal solutions were synthesized by the same method as NiBHT (Figure 2b). No precipitation formed and homogeneous dispersions were maintained for at least 2 weeks (Figure S1, Supporting Information). The UV–vis–NIR spectra of CuBHT and ZnBHT colloidal solutions measured under Ar. The CuBHT colloidal solution showed an absorption peak in the visible to near‐infrared region, while the ZnBHT solution showed an absorption peak at 280 nm originating from the ligand site π–π^*^ transition (Figure S10, Supporting Information). CuBHT and ZnBHT obtained by the liquid‐liquid interfacial synthesis have been reported to have conductive and insulating natures, respectively.^[^ ^34^, ^35^, ^36^, ^56^ ^]^ The observed absorption of CuBHT suggests a small band gap and metallic properties, while the absorption of ZnBHT at the UV region indicates a large band gap, which is consistent with the results of previous studies. The Raman spectrum of CuBHT corresponds to that of CuBHT obtained by liquid‐liquid interfacial synthesis.^[^ ^34^ ^]^ The Raman spectrum of ZnBHT measured in an inert atmosphere was consistent with that of ZnBHT obtained by the interfacial reaction. Hence, colloidal MBHT solutions (M = Cu, Zn) were successfully prepared by a single‐phase synthesis method (Figure S11, Supporting Information).

### 2.2 Sequential Synthesis of NiBHT from NiDT

The structure of NiBHT can be regarded as having the pores of NiDT filled with six Ni^2+^ ions and one BHT molecule. Therefore, we expect that NiBHT can be obtained by adding Ni^2+^ ions to a colloidal NiDT solution that includes excess BHT. At first, we measured time‐dependent UV–vis–NIR spectroscopy of Ni_1_/BHT colloidal solution to investigate the reaction time required to complete NiDT formation under Ar. The absorbance of the characteristic peak at ≈970 nm of NiDT gradually increased with the reaction time and reached a constant value after 6 h (Figure 2l,m; Figure S12, Supporting Information), indicating that NiDT formation in Ni_1_/BHT colloidal solution was completed after 6 h. When Ni(OAc)_2_ solution was added to the Ni_1_/BHT colloidal solution after 6 h, the color of the solution became darker. Time‐dependent UV–vis–NIR spectroscopy of Ni_1_/BHT after the addition of Ni ions exhibited that the absorption peak at ≈970 nm of NiDT immediately shifted to ≈1090 nm and the reaction was completed in 2.5 h (Figure S13, Supporting Information). The UV–vis–NIR and Raman spectra of NiDT+Ni were consistent with those of NiBHT prepared by the one‐step, single‐phase reaction method described above (Figure S14, Supporting Information). Hence, NiBHT can be synthesized by sequentially adding nickel salt to a NiDT solution, including excess BHT.

### 2.3 Sequential Synthesis of NiM_2_BHT (M = Cu, Zn)

**Figure 3:**
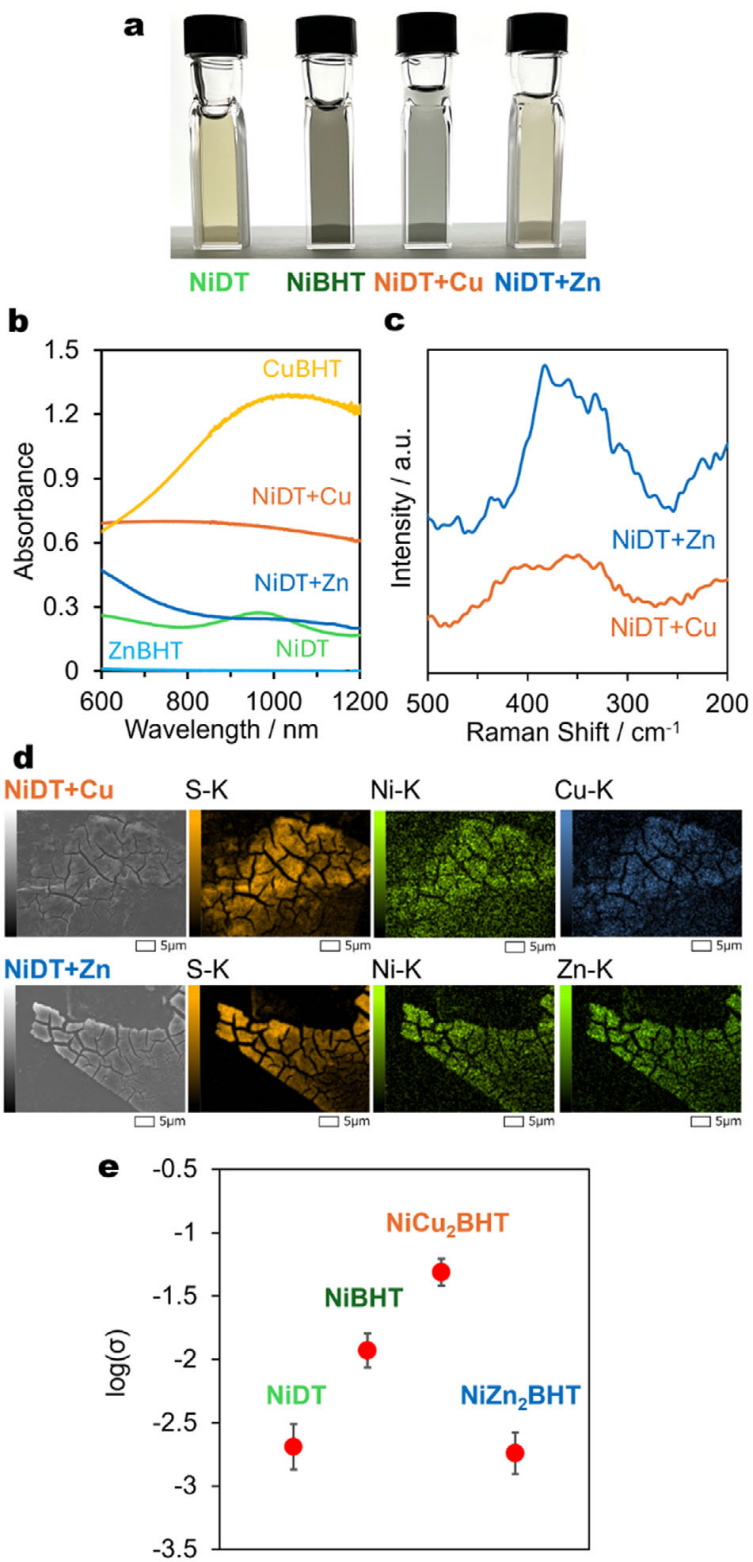
Synthesis and electrical conductivity of heterometallic coordination nanosheets. a), Photo of NiDT, NiBHT, NiDT+Cu, and NiDT+Zn colloidal solutions. b), vis–NIR absorption spectra of NiDT, CuBHT, NiDT+Cu, ZnBHT, and NiDT+Zn colloidal solutions. c), Raman spectra of NiDT+Cu under air and NiDT+Zn under Ar. d), SEM‐EDS elemental mappings of NiDT+Cu and NiDT+Zn. e), Electrical conductivity of pelletized NiDT, NiBHT, NiCu_2_BHT, and NiZn_2_BHT at room temperature.

Furthermore, we applied this sequential synthesis method using NiDT to produce heterometallic coordination nanosheets, NiM_2_BHT. When the Cu(OAc)_2_ solution was added to the NiDT colloidal solution, the color of the dispersion solution turned bluish black (**Figure** **3**a). No precipitation formed and homogeneous dispersions were maintained for at least 2 weeks (Figure S1, Supporting Information). The UV–vis–NIR spectrum of NiDT+Cu showed a peak at ≈850 nm and the disappearance of a peak at ≈970 nm in NiDT. This spectrum differs from CuBHT, suggesting that adding Cu^2+^ ion did not progress CuBHT formation (Figure 3b). Furthermore, the Raman spectrum of NiDT+Cu was consistent with that of NiCu_2_BHT obtained by the interfacial reaction reported in a previous study (Figure 3c).^[^ ^34^ ^]^ SEM‐EDS and STEM‐EDS mapping showed a uniform distribution of S, Ni, and Cu on NiDT+Cu (Figure 3d; Figure S15a, Supporting Information). Furthermore, the elemental ratio of S: Ni: Cu on 7.9: 1: 1.9 is roughly matched with the predicted ratio (S: Ni: Cu = 6: 1: 2). This series of results indicates that NiCu_2_BHT can be sequentially synthesized by adding Cu^2+^ ions after NiDT formation.

The NiDT colloidal solution turned lighter brown by adding a Zn(OAc)_2_ solution (Figure 3a). No precipitation formed and homogeneous dispersions were maintained for at least 2 weeks (Figure S1, Supporting Information). The UV‐vis–NIR spectrum of NiDT+Zn showed a peak at ≈1050 nm and the disappearance of a peak at ≈970 nm in NiDT. This spectrum differs from ZnBHT, suggesting the formation of a novel nanosheet (Figure 3b). The Raman spectrum of NiDT+Zn under Ar exhibited a peak at 383 cm^−1^ not observed in the spectra of NiDT and ZnBHT (Figure 3c). SEM‐EDS and STEM‐EDS mapping showed a uniform distribution of S, Ni, and Zn on NiDT+Zn (Figure 3d; Figure S15b, Supporting Information). Furthermore, the elemental ratio of S: Ni: Zn on 6.6: 1: 1.6 is roughly correlated with the predicted value (S: Ni: Zn = 6: 1: 2). This series of results indicates that we successfully achieved the synthesis of a novel heterometallic coordination nanosheet, NiZn_2_BHT by sequential addition of Zn^2+^ ions to the NiDT colloidal solution.

### 2.4 Electrical Conductivity

The electrical conductivity of pelletized NiDT, NiBHT, NiCu_2_BHT, and NiZn2BHT (Figure 2c) was measured by the van der Pauw method. Their electrical conductivity at room temperature was 2.2 × 10^−3^, 1.2 × 10^−2^, 5.0 × 10^−2^, and 2.0 × 10^−3^ S cm^−1^, respectively, which shows that the addition of metal to NiDT leads to different electrical conductivity (Figure 3e). The electrical conductivity of NiDT, NiBHT, and NiCu_2_BHT obtained by the interface synthesis method in the previous study was 2.8, 90, and 660 S cm^−1^, respectively, which was consistent with the tendency of the coordination nanosheet mentioned above by the single‐phase synthesis method.^[^ ^26^, ^34^ ^]^ Since Zn^2+^ has a closed shell structure of d^10^, the reaction of NiDT with Zn^2+^ could not affect electrical conductivity, resulting in little change in electrical conductivity from NiDT to NiZn_2_BHT. On the other hand, the electrical conductivity of the coordination nanosheets synthesized by a single‐phase reaction was 3–4 orders of magnitude smaller than that of the interfacial reaction. This difference is attributed to the fragment size being smaller than that of the film, which leads to greater total resistance due to the extrinsic resistance between fragments.

### 2.5 Transmetallation Reaction of NiBHT

**Figure 4:**
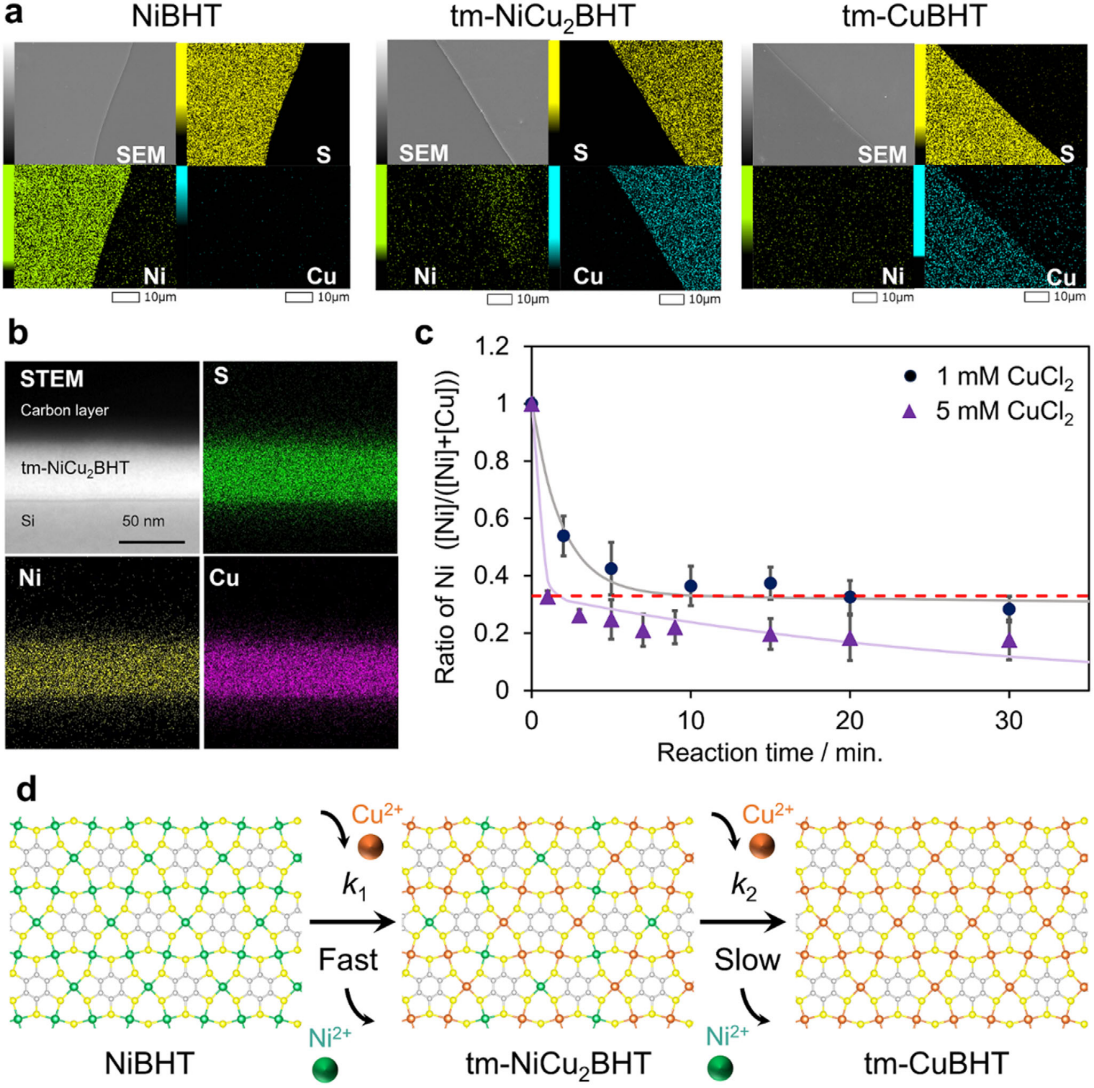
Transmetallation reaction of NiBHT film. a) SEM‐EDS elemental mappings of NiBHT, tm‐NiCu_2_BHT, and tm‐CuBHT. b) Cross‐sectional STEM‐EDS elemental mapping of tm‐NiCu_2_BHT. c) Kinetic analysis of the transmetallation process. The red dashed line shows 1/3, corresponding to the ratio of Ni in NiCu_2_BHT. d) Stepwise transmetallation process from NiBHT to tm‐CuBHT via metastable tm‐NiCu_2_BHT.

Transmetallation is a technique that allows metal ions to be exchanged while maintaining the structure of the original coordination nanosheet.^[^ ^56^, ^62^, ^63^, ^64^ ^]^ During the transmetallation reaction, a heterometallic coordination nanosheet can be formed. We investigated whether NiCu_2_BHT is formed in the transmetallation reaction from NiBHT to CuBHT. A thin film of NiBHT with a thickness of ca. 100 nm was prepared by liquid‐liquid interfacial synthesis. It was drop‐cast onto a silicon substrate and the sample was immersed in a 5 mm CuCl_2_ aqueous solution for 48 h to synthesize tm‐CuBHT. First, SEM/EDS measurements were performed to confirm the metal abundance ratio. In NiBHT before immersion, only Ni was detected as a metal and uniformly distributed together with S (**Figure** **4**a left; Figure S16a, Supporting Information). After immersion in CuCl_2_ aq., Cu was instead detected in the tm‐CuBHT, and the Ni: Cu ratio became 4: 96 (Figure 4a right; Figure S16c, Supporting Information). Furthermore, chlorine as counter anion was not detected after the reaction. Therefore, the detection of Cu was attributed to transmetalltion from Ni to Cu, not caused by adsorption of CuCl_2_ ion pairs. These results indicate that the transmetallation reaction from Ni to Cu had progressed with a yield of 96%. Furthermore, by shortening the reaction time to 8 min, a heterometallic nanosheet tm‐NiCu_2_BHT was obtained (Figure 4a center; Figure S16b, Supporting Information). Its Ni: Cu ratio was 31: 69, which was close to that of the previously reported NiCu_2_BHT. The transmetallation reaction was also confirmed by XPS (Figures S17–S19, Supporting Information). In the Raman spectrum, peaks ascribed to the M─S bond were observed at different wavenumbers, indicating that tm‐NiCu_2_BHT is a single phase and not a mixture of NiBHT and CuBHT. Furthermore, the spectrum of tm‐NiCu_2_BHT is similar to that of NiCu_2_BHT in the previous report and described above (Figure S20, Supporting Information).^[^ ^34^ ^]^ The fact that tm‐NiCu_2_BHT is a single phase was also confirmed by cross‐sectional STEM‐EDS elemental mapping. S, Ni, and Cu were uniformly distributed in the thin film (Figure 4b). The elemental ratio of S: Ni: Cu ions remained 8.3: 1: 2.1. From the above results, heterometallic NiCu_2_BHT nanosheet can be produced using the transmetallation reaction. In contrast, the metal exchange yield was only 14% after 72 h in the transmetallation of CuBHT using NiCl_2_, indicating that the transmetallation from Cu to Ni is slower than that from Ni to Cu (Figure S21, Supporting Information).

Next, the reaction kinetics of the transmetallation reaction was then analyzed by changing the concentration of the CuCl_2_ solution to 1 and 5 mm. Figure 4c shows the ratio of nickel to the total metal species, [Ni]/([Ni]+[Cu]), obtained by SEM‐EDS versus the transmetallation reaction time. In the reaction with 1 mm CuCl_2_ aq., the Ni ratio reached ≈0.33 at 20 min and slowly decreased after that. This indicates a two‐step reaction through the metastable phase NiCu_2_BHT (Figure 4d). In the reaction with 5 mm CuCl_2_ aq., it was seen that the reaction proceeded more quickly. We assumed a two‐step first‐order reaction as follows.

(1) $$\mathrm{NiBHT}\;\overset{k_{1}}{\to }\;\mathrm{tm}-\mathrm{NiC}u_{2}\mathrm{BHT}\;\overset{k_{2}}{\to }\;\mathrm{tm}-\mathrm{CuBHT}$$

The reaction rate constants for each step *k*_1_ and *k*_2_ were calculated to be 5.3 × 10^−1^ and 2 × 10^−3^ mm ^−1^ min^−1^, respectively (See Method for details). From this result, it was found that the reaction proceeded as a two‐step first‐order reaction, and *k*_2_ was two orders of magnitude smaller than *k*_1_. To demonstrate that tm‐NiCu_2_BHT is a metastable phase, liquid‐liquid interfacial synthesis was performed at 80 °C. At room temperature, NiCu_2_BHT was formed at the interface between a BHT dichloromethane solution and an aqueous solution of Ni: Cu = 1: 2. On the other hand, at the interface between a chlorobenzene solution of BHT and an aqueous solution of Ni: Cu = 1: 2 at 80 °C, CuBHT was formed at the interface (Figure S22, Supporting Information). This result shows that Ni in NiCu_2_BHT is replaced by Cu at high temperatures, indicating that NiCu_2_BHT is a metastable phase.

Finally, we investigated the electrical conductivity of NiBHT, tm‐NiCu_2_BHT, and tm‐CuBHT to reveal whether this heterometallic nanosheet exhibits properties different from those of a homometallic nanosheet. It is known that NiCu_2_BHT, a coordination nanosheet obtained by mixing nickel and copper ions and reacting them with BHT, exhibits higher electrical conductivity than homometallic nanosheets. Therefore, in this study, the electrical conductivity of tm‐NiCu_2_BHT synthesized by a transmetallation reaction was measured using the two‐terminal method using interdigitated array electrodes (IDA). The transmetallation was carried out using NiBHT‐modified on IDA (Figure S23a, Supporting Information). The conductivity measurements for NiBHT, tm‐NiCu_2_BHT, tm‐CuBHT were conducted using the same flake of the nanosheets. The electrical conductivity of tm‐NiCu_2_BHT was 3.2 × 10^−1^ S cm^−1^, which is higher than that of the homometallic NiBHT and tm‐CuBHT (Figure S23b, Supporting Information), which is consistent with previously reported results.^[^ ^34^ ^]^ On the other hand, the electrical conductivity is smaller than the previously reported values. The reason for this is probably related to the larger resistance between fragments in the film and small cracks caused under the experiment, which reduced the actual electron path through the film. Despite these experimental difficulties, the comparison of a series of coordination nanosheet films before and after the transmetallation using the same samples clearly indicated the increase of electrical conductivity in the mixed‐metal phase.

## 3 Conclusion

In this study, we established two novel methods for the synthesis of heterometallic NiM_2_BHT (M = Cu, Zn) with a regular structure. The first method is based on the preparation of a colloidal solution by a single‐phase reaction of metal ions with BHT. It was confirmed by XAFS, UV–vis–NIR, and Raman spectroscopy that the selective synthesis of two structures, NiDT and NiBHT, can be achieved from a stoichiometric approach. Thanks to their colloidal solution, HER electrode can be easily produced by coating. NiDT was the better catalyst than NiBHT probably due to the pores. After NiDT synthesis, NiCu_2_BHT was produced by adding Cu^2+^ ions and NiZn_2_BHT by adding Zn^2+^ ions. The structures were identified by UV–vis–NIR, SEM‐EDS, and Raman spectroscopy. The structure of NiCu_2_BHT was confirmed to be consistent with that obtained by the two‐phase interfacial synthesis method. The electrical conductivity of NiDT, NiBHT, NiCu_2_BHT, and NiZn_2_BHT obtained by the single‐phase synthesis method was NiCu_2_BHT > NiBHT > NiDT ≈ NiZn_2_BHT, which was consistent with the trend of electrical conductivity values of NiDT, NiBHT, and NiCu_2_BHT obtained by the interfacial synthesis method.

The second method is based on the transmetallation reaction from NiBHT to CuBHT, and kinetic analysis confirmed that it proceeds in two steps. The metastable product of the first step reaction was confirmed to be tm‐NiCu_2_BHT with the composition NiCu_2_BHT. Identification was performed by AFM, SEM‐EDS, GIXS, cross‐sectional STEM‐EDS, Raman spectroscopy, and XPS, and it was confirmed that tm‐NiCu_2_BHT has a single‐phase structure. A kinetic analysis of the two‐step reaction was performed based on the relationship between reaction time and metal element ratio, and it was found that the reaction rate constants for the transmetallation from NiBHT to tm‐NiCu_2_BHT and from tm‐NiCu_2_BHT to tm‐CuBHT were *k*_1_ = 5.3 × 10^−1^ and *k*_2_ = 2.1 × 10^−3^ mm ^−1^ min^−1^, respectively. In addition, the electrical conductivity of each coordination nanosheet obtained by the transmetallation reaction was measured and it was confirmed that the electrical conductivity of tm‐NiCu_2_BHT exceeded that of NiBHT and tm‐CuBHT.

In addition to the previously reported synthesis of NiCu_2_BHT, this study achieved the synthesis of a new heterometallic coordination nanosheet, NiZn_2_BHT. The single‐phase colloidal solution method with porous MDT makes it possible to create more diverse heterometallic coordination nanosheets.

## Conflict of Interest

The authors declare no conflict of interest.

## Supporting information



Supporting Information

## Data Availability

The data that support the findings of this study are available from the corresponding author upon reasonable request.
