# Supplementary material for: Rationally Engineered Heterometallic Metalladithiolene Coordination Nanosheets with Defined Atomic Arrangements
Source: Small. 2025 May 5;21(38):2503227. doi: 10.1002/smll.202503227 (PMC12462590; doi:10.1002/smll.202503227)
Supplement: Supplementary file 1 — Supporting Information [file SMLL-21-2503227-s001.pdf]

NANO · MICRO
small

Supporting Information

for *Small*, DOI 10.1002/smll.202503227

Rationally Engineered Heterometallic Metalladithiolene Coordination Nanosheets with Defined Atomic Arrangements

*Miyu Ito, Naoya Fukui, Kenji Takada, Ziheng Yu, Hiroaki Maeda, Katsuya Mizuno and Hiroshi Nishihara**

Supporting Information

Rationally Engineered Heterometallic Metalladithiolene Coordination Nanosheets with Defined Atomic Arrangements

Miyu Ito[1], Naoya Fukui[2], Kenji Takada[2], Ziheng Yu[1], Hiroaki Maeda[2], Katsuya Mizuno[2], Hiroshi Nishihara[1,2]*

[1] Graduate School of Science and Technology, Tokyo University of Science, 2641 Yamazaki, Noda, Chiba 278-8510, Japan

[2] Research Institute for Science and Technology, Tokyo University of Science, 2641 Yamazaki, Noda, Chiba 278-8510, Japan

*Corresponding authors. E-mail addresses: nisihara@rs.tus.ac.jp

Content

Materials

Apparatus

References

Figure S1. Ni_x/BHT colloidal solutions (x = 1,3), CuBHT, ZnBHT, NiDT+Cu and NiDT+Zn colloidal solution of 2 weeks after synthesis.

Figure S2. Temporal change in the particle size of Ni_x/BHT.

Figure S3. TEM image and particle size frequency obtained from TEM image of Ni_x/BHT.

Figure S4. UV–Vis–NIR absorption spectrum of NiBHT synthesized by the liquid-liquid interfacial reaction.

Figure S5. Time-dependent UV-Vis-NIR absorption spectra of Ni_1/BHT under air from 0 to 1.5 h.

Figure S6. Raman spectra of NiDT without unreacted BHT.

Figure S7. XP spectra of Ni_1/BHT and Ni_3/BHT.

Figure S8. Electrochemical measurement of NiDT and NiBHT.

Figure S9. Electrocatalytic performance of NiDT and NiBHT.

Figure S10. UV–Vis–NIR absorption spectra of MBHT (M= Cu, Zn).

Figure S11. Raman spectra of MBHT.

Figure S12. Time-dependent UV-Vis-NIR absorption spectra of Ni_1/BHT from 0 to 6 h.

Figure S13. Vis-NIR absorption spectra during the reaction from NiDT to NiBHT.

Figure S14. Raman spectra of NiDT, NiBHT, and NiDT+Ni.

Figure S15. STEM-EDS of NiM_2BHT.

Figure S16. SEM-EDS of MBHT films.

Figure S17. XP spectrum of NiBHT film.

Figure S18. XP spectrum of tm-$NiCu_2BHT$.

Figure S19. XP spectrum of tm-CuBHT.

Figure S20. Raman spectra of MBHT films.

Figure S21. SEM-EDS of transmetallated CuBHT film with $NiCl_2$.

Figure S22. SEM-EDS of $NiCu_2BHT$ prepared at 80 °C.

Figure S23. Relations between electrical conductivity and ratio of transmetallated Cu^{2+} ions.

Methods

Materials

BHT and ZnBHT were synthesized according to the literatures [S1, S2]. $Ni(OAc)_2 \cdot 4H_2O$, $Cu(OAc)_2 \cdot H_2O$, $CuCl_2 \cdot 2H_2O$, anhydrous THF, methanol (HPLC grade), and dichloromethane (HPLC grade) were purchased from KANTO CHEMICAL CO., INC. $NiCl_2 \cdot 6H_2O$ and $Zn(OAc)_2 \cdot 2H_2O$ were obtained from FUJIFILM Wako Pure Chemical Corporation. These commercially available chemicals were used without further purification. The water was purified with an Autopure WD500 system (Yamato Scientific Co., Ltd.).

Apparatus

UV-Vis-NIR spectra were recorded with a JASCO V-770 spectrometer equipped with an integral sphere unit ILS-923 using MBHT colloidal solution filled in a 1 cm quartz cell. DLS was measured with a HORIBA SZ-100V2. SEM-EDS were collected with a JEOL JCM-7000 electron microscope with an acceleration voltage of 15 kV. Samples for SEM observation were prepared by the deposition of colloidal MBHT solutions or films on Si substrates by dropcasting. TEM measurements were performed for samples on elastic carbon film-coated Cu grids using a JEOL-2100F electron microscope with an acceleration voltage of 200 kV. STEM/EDS measurements were performed for samples on carbon film-coated Mo grids using a JEM-ARM200F (JEOL Ltd.) with an acceleration voltage of 200 kV. Samples for cross-sectional TEM measurements were fabricated with a Hitachi XVision 200T FIB-SEM operated at a 30 kV Ga ion beam. Raman spectroscopy was performed with a JASCO NRS-5500 spectrometer under 532 nm laser irradiation. Samples for Raman spectroscopy were deposited on Si substrates under an Ar atmosphere. A sealed cell prepared in an Ar-filled glove box was used for spectroscopy under an inert atmosphere. XPS was measured using a PHI 5000 VersaProbe or PHI VersaProbeIII spectrometer. Al Kα (15 kV, 25 W) radiation was used as an X-ray source. The sample was deposited on a piece of graphitic carbon paper. The spectra were analyzed using Multi Pak Software, and the binding energy was standardized using a C 1s peak at 284.6 eV. The AFM images were collected under

ambient conditions using an Agilent Technologies 5500 scanning probe microscope with an NCH silicon cantilever (Nano World) in AC mode and a NaioAFM with a PPP-NCLR probe in AC mode. Ni_x/BHT and $NiCu_2BHT$ for AFM measurements were ultrasonicated for 1 h and then spincoated with ACT-220 DII spincoater (ACTIVE Co., Ltd.) on SiO_2/Si substrates (1×1 cm^2) with 3000 rpm for 5 s.

Preparation of Ni_x/BHT colloidal solutions

Under Ar, $Ni(OAc)_2$ in MeOH (10 mL, 0.15 mM. 0.225 mM, 0.30 mM or 0.45 mM) was added to a solution of BHT in THF (10 mL, 0.15 mM). The mixture was vigorously stirred for 1 min.

Interfacial synthesis of NiBHT

We synthesised NiBHT according to the literature with slight modification [34]. Under Ar, 11.1 mg of BHT was dissolved in CH_2Cl_2 (35 mL) to prepare a saturated BHT solution. After removing insoluble contaminants by filtration, the BHT solution was diluted by adding CH_2Cl_2 (35 mL). In a 50 mL vial with quartz substrate at the bottom, 10 mL of H_2O was layered on the BHT solution (10 mL) to form H_2O/CH_2Cl_2 interface. To the aqueous phase, an aqueous solution of $Ni(OAc)_2$ (10 mL, prepared by dissolving 50.5 mg of $Ni(OAc)_2{\cdot}4H_2O$ in 100 mL of H_2O) was slowly added and the reaction container was kept calm for 90 min. The resulting black NiBHT film was deposited on the quartz substrate by removing the dichloromethane phase, and the NiBHT was washed with MeOH.

Preparation of CuBHT colloidal solution

Under Ar, $Cu(OAc)_2$ in MeOH (10 mL, 0.45 mM) was added to a solution of BHT in THF (10 mL, 0.15 mM). The mixture was vigorously stirred for 1 min.

Preparation of ZnBHT colloidal solution

Under Ar, $Zn(OAc)_2$ in MeOH (10 mL, 0.45 mM) was added to a solution of BHT in THF (10 mL, 0.15 mM). The mixture was vigorously stirred for 1 min.

Preparation of NiBHT colloidal solution

Under Ar, $Ni(OAc)_2$ in MeOH (10 mL, 0.15 mM) was added to a solution of BHT in THF (10 mL, 0.15 mM). The mixture was vigorously stirred for 1 min. The mixture was kept calm for 6 h. Then, $Ni(OAc)_2$ in MeOH (200 μL, 15 mM) was added and vigorously stirred for 1 min and the mixture was kept calm for another 2.5 h.

Preparation of $NiCu_2BHT$ colloidal solution

Under Ar, $Ni(OAc)_2$ in MeOH (10 mL, 0.15 mM) was added to a solution of BHT in THF (10 mL, 0.15 mM). The mixture was vigorously stirred for 1 min. The mixture was kept calm for 6 h. Then, $Cu(OAc)_2$ in MeOH (200 μL, 15 mM) was added and vigorously stirred for 1 min and the mixture was kept calm for another 2.5 h.

Preparation of $NiZn_2BHT$ colloidal solution

Under Ar, $Ni(OAc)_2$ in MeOH (10 mL, 0.15 mM) was added to a solution of BHT in THF (10 mL, 0.15 mM). The mixture was stirred vigorously for 1 min. The mixture was kept calm for 6 h. Then, $Zn(OAc)_2$ in MeOH (200 μL, 15 mM) was added vigorously stirred for 1 min and the mixture was kept calm for another 2.5 h.

Collection of coordination nanosheets colloids as powder

As prepared colloidal was concentrated by evaporation of solvent until the volume was reduced to 1/30. Then hexane (100 mL) was added, and the formed powder was filtrated, washed with MeOH and THF, dried under vacuum.

Preparation of NiBHT films for transmetallation reaction

Under Ar , a saturated solution of BHT in CH_2Cl_2 was diluted by ten times. In a 20 mL vial, pure water (5 mL) was layered on the diluted BHT solution to form liquid-liquid interface. A solution of 10 mM $Ni(OAc)_2$ (5 mL) was added to the aqueous phase to start the reaction. After 24 h, the aqueous phase was diluted with pure water several times to stop the reaction. All the solvents were removed from the bottom and pure ethanol was added to the residual thin films.

Preparation of CuBHT films for transmetallation reaction

Under Ar, a saturated solution of BHT in CH_2Cl_2 was diluted ten times. In a 20 mL vial, pure water (5 mL) was layered on the diluted BHT solution to form the liquid-liquid interface. A solution of 10 mM $Cu(OAc)_2$ (5 mL) was added to the aqueous phase to start the reaction. After 24 h, the aqueous phase was diluted with pure water several times to stop the reaction. All the solvents were removed from the bottom and pure ethanol was added to the residual thin films.

Transmetallation of NiBHT

NiBHT films floating in ethanol were drop-casted on Si substrates. The NiBHT-decorated substrates were immersed in an aqueous solution of $CuCl_2$ (5 mM, 5 mL). After the reaction, the substrates were washed with pure water and ethanol several times and dried.

Transmetallation of CuBHT

CuBHT films floating in ethanol were drop-casted on Si substrates. The CuBHT-decorated substrates were immersed in an aqueous solution of $NiCl_2$ (5 mM, 5 mL). After the reaction, the substrates were washed with pure water and ethanol several times and dried.

XANES measurement

Synchrotron-radiated XANES were measured at the NanoTerasu BL08W beamline. Pelletized Ni_x/BHT was measured by transmission and fluorescence methods for the Ni K-edge and S K-edge spectra, respectively. Obtained spectra were analyzed with the Athena and Artemis softwares.

Electrochemical measurement

Electrochemical measurements were performed using an ALS 750E potentiostat (BAS) and RRDE-3A rotating ring disk electrode apparatus (BAS). Glassy carbon (GC) rotating disk electrodes (3 mmϕ) modified with Ni_x/BHT (x = 1, 3) were prepared by dropping 10 μL of Ni_x/BHT colloidal solutions on alumina-polished electrodes, then drying under vacuum before use as working electrodes. A Pt wire was used as a counter electrode.

Cyclic voltammetry to investigate the redox properties and capacitance of Ni_x/BHT was conducted using a conventional three-electrode cell set up with a 0.1 M $^nBu_4NPF_6/CH_2Cl_2$ solution under Ar. The potential was recorded with an Ag^+/Ag reference electrode (a silver wire immersed in a 0.1 M $AgClO_4$/0.1 M $^nBu_4NPF_6/CH_3CN$ solution) and calibrated with the redox potential of ferrocenium/ferrocene (Fc^+/Fc, 0.242 V vs. Ag^+/Ag). The electric double layer capacitances (C_{dl}) of Ni_x/BHT were calculated as the following equation:

$$\frac{I_a - I_c}{2} = C_{dl}v$$

Where I_a and I_c are the anodic and cathodic currents at −0.842 V vs. Fc^+/Fc, respectively, and v is a scan rate.

The evaluation of electrocatalytic performance was conducted with a degassed 0.5 M H_2SO_4 aqueous solution (pH 0.42 – 0.48) as the electrolyte solution. The pH values of electrolyte solutions were measured by a HORIBA D-55S pH meter. A reversible hydrogen electrode (RHE) was used as a reference electrode. Linear sweep voltammetry was performed at the scan rate of 10 mV s^{-1} and the rotational rate of 1600 rpm at room

temperature. The voltammograms of Pt and non-modified GC rotating electrodes were also collected under the same condition.

Kinetic analysis

To analyze the reaction kinetics of the transmetallation of NiBHT, we assumed the following two-step reaction,

$$\text{NiBHT} \overset{k_1}{\rightarrow} \text{tm-NiCu}_2\text{BHT} \overset{k_2}{\rightarrow} \text{tm-CuBHT}$$

where k_1 and k_2 are reaction rate constant for each step. The reaction rate were obtained by solving following equations.

$$-\frac{d[\text{NiBHT}]}{dt} = k_1[\text{NiBHT}][\text{CuCl}_2]$$

$$-\frac{d[\text{tm-NiCu}_2\text{BHT}]}{dt} = k_2[\text{tm-NiCu}_2\text{BHT}][\text{CuCl}_2] - k_1[\text{NiBHT}][\text{CuCl}_2]$$

$$\frac{d[\text{tm-CuBHT}]}{dt} = k_2[\text{tm-NiCu}_2\text{BHT}][\text{CuCl}_2]$$

The concentration of $CuCl_2$ was high enough to be considered as constant. Therefore, The ratio of nickel to the total metals was given as follows.

$$\frac{[\text{Ni}]}{[\text{Ni}] + [\text{Cu}]} = e^{-k_{1app}t} + \frac{k_{1app}}{3(k_{2app} - k_{1app})}(e^{-k_1 appt} - e^{-k_2 appt})$$

$k_{1\text{app}}$ and $k_{2\text{app}}$ were defined as following.

$k_{1app} = k_1[CuCl_2]$

$k_{2app} = k_2[CuCl_2]$

The fitting of experimental data gave k_{1app} and k_{2app} for each $CuCl_2$ concentration.

I-V measurements for MBHT

I-V measurements for MBHT were collected by the van der Pauw method. Four probes were contacted to pelletised samples (ϕ 4.5 mm) with carbon paste. The thicknesses of the pellets were measured with a micrometre.

I-V measurements for NiBHT, tm-$NiCu_2$BHT, and tm-CuBHT films

Electric conductivity measurement for MBHT films was performed by the two probe method with Au interdigitated array electrodes (GMT-AU10/5, GEOMATEC), the gap between the two electrodes and the width of the electrodes were 5 and 10 μm, respectively. A flake of NiBHT was drop-casted on an IDA and dried under vacuum. First, the conductivity of NiBHT was measured. Then, the transmetallation was performed by immersing the decorated IDA in 5 mM $CuCl_2$ solutions to form tm-$NiCu_2$BHT, and its conductivity was measured. tm-$NiCu_2$BHT was further transmetallated by immersing the sample in a 5 mM $CuCl_2$ solution again to form tm-CuBHT and its conductivity was measured.

References

S1) Harnisch, J. A.; Angelici, R, J. Gold and platinum benzenehexathiolate complexes as large templates for the synthesis of 12-coordinate polyphosphine macrocycles. *Inorg. Chim. Acta* **2000**, *300–302*, 273–279.

S2) Tan, C. M.; Fukui, N.; Takada, K.; Maeda, H.; Selezneva, E.; Bourgès, C.; Masunaga, H.; Sasaki, S.; Tsukagoshi, K.; Mori, T.; Sirringhaus, H.; Nishihara, H. Lateral Heterometal Junction Rectifier Fabricated by Sequential Transmetallation of Coordination Nanosheet. *Angew. Chem. Int. Ed.* **2024**, *63* (9), e202318181.

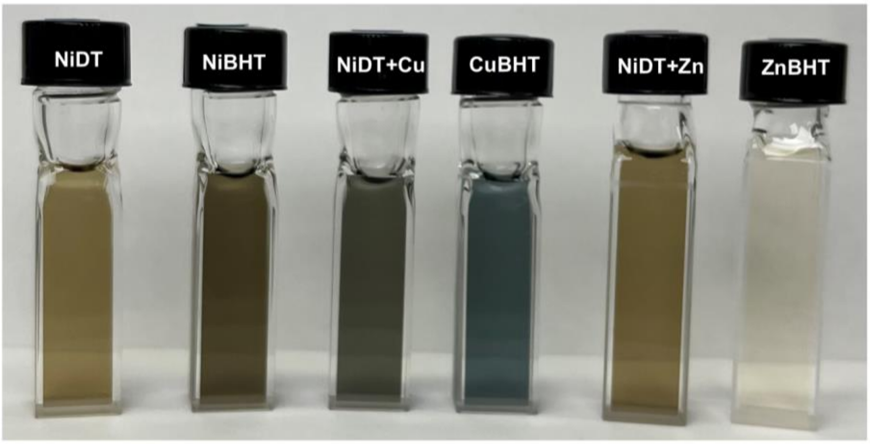

Figure S1. Ni_x/BHT colloidal solutions (*x* = 1,3), CuBHT, ZnBHT, NiDT+Cu and NiDT+Zn colloidal solution of 2 weeks after synthesis.

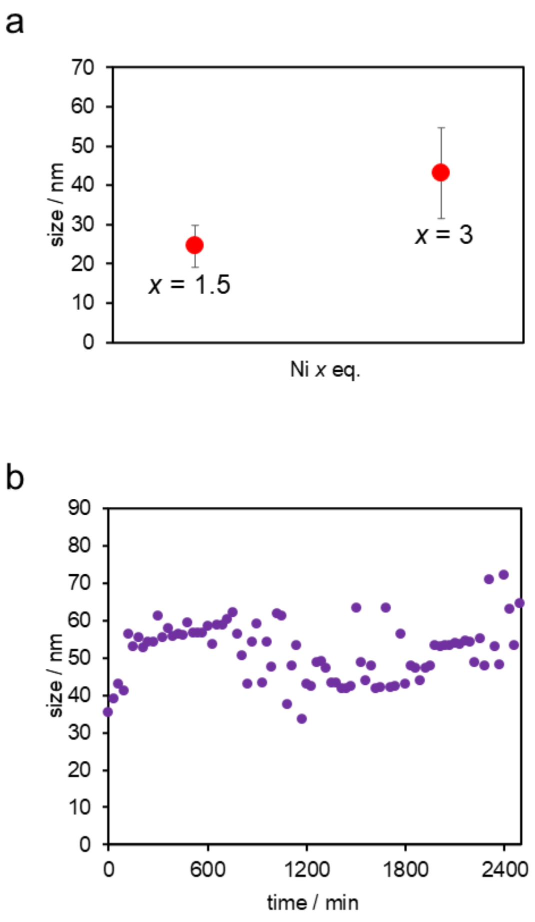

Figure S2. Temporal change in the particle size of Ni_x/BHT. a, DLS particle size of Ni_x/BHT (x =1.5, 3). **b,** Time-dependence of particle size of Ni_3/BHT from 0 to 2500 min measured by DLS.

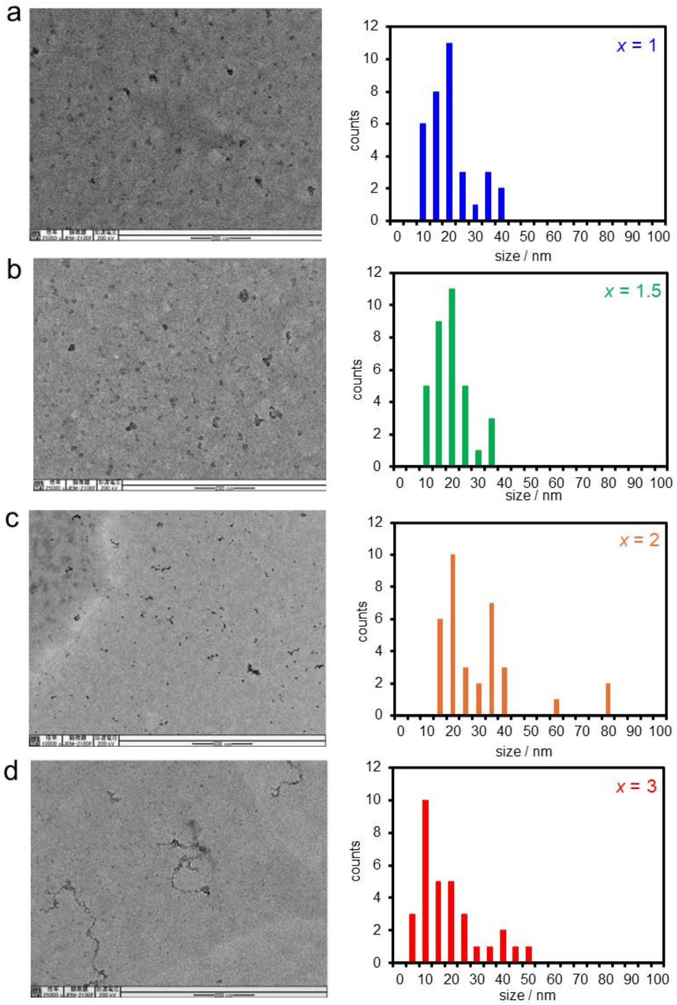

Figure S3. TEM image and particle size frequency obtained from TEM image of Ni$_x$/BHT. a, x =1. **b,** x =1.5. **c,** x =2. **d,** x =3.

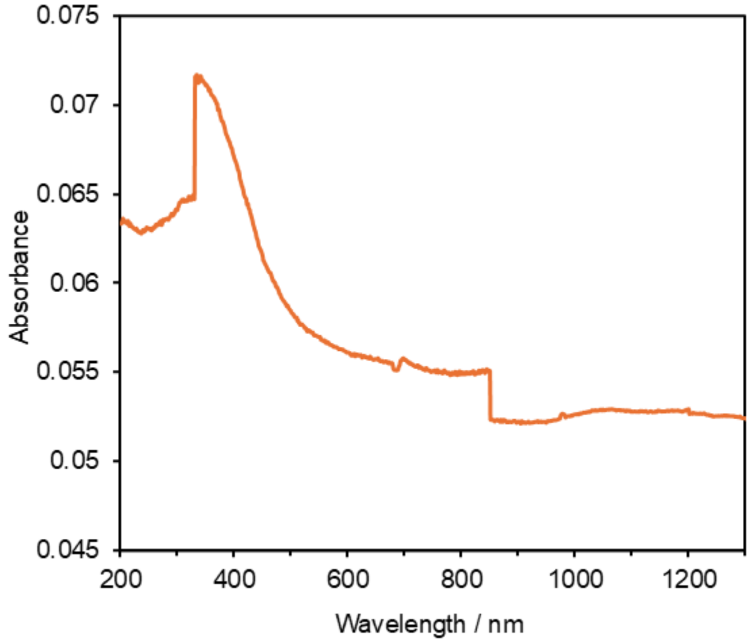

Figure S4. UV–Vis–NIR absorption spectrum of NiBHT synthesized by the liquid-liquid interfacial reaction.

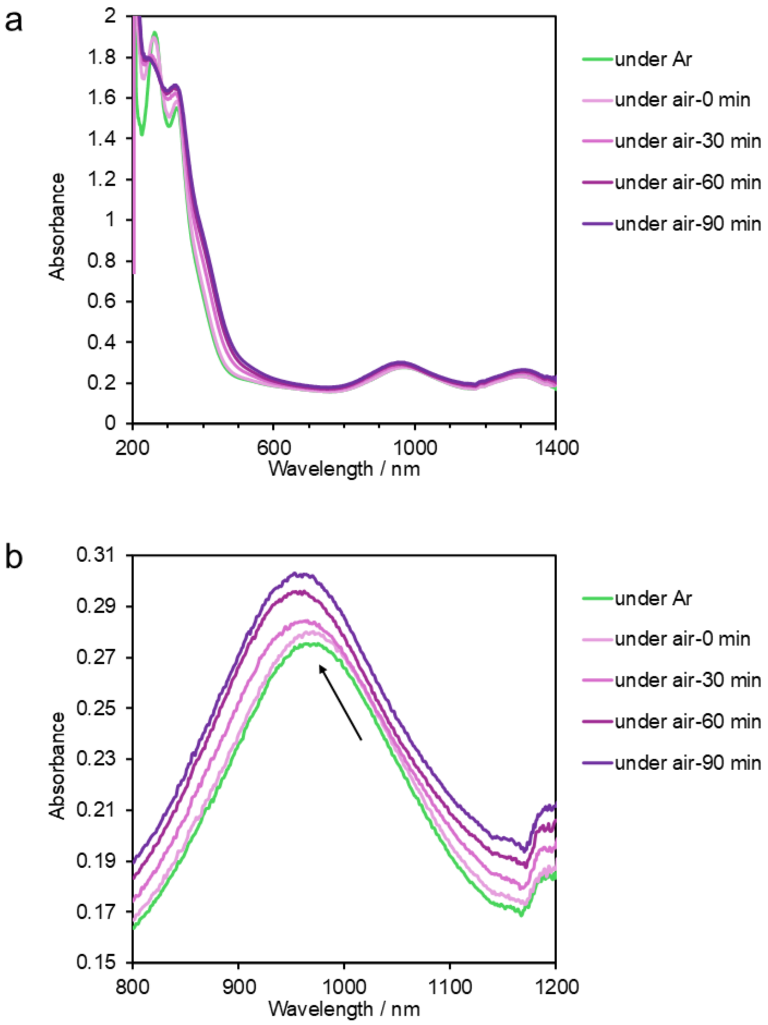

Figure S5. Time-dependent UV-Vis-NIR absorption spectra of Ni_1/BHT under air from 0 to 1.5 h. a, 200-1400 nm. **b,** 800-1200 nm.

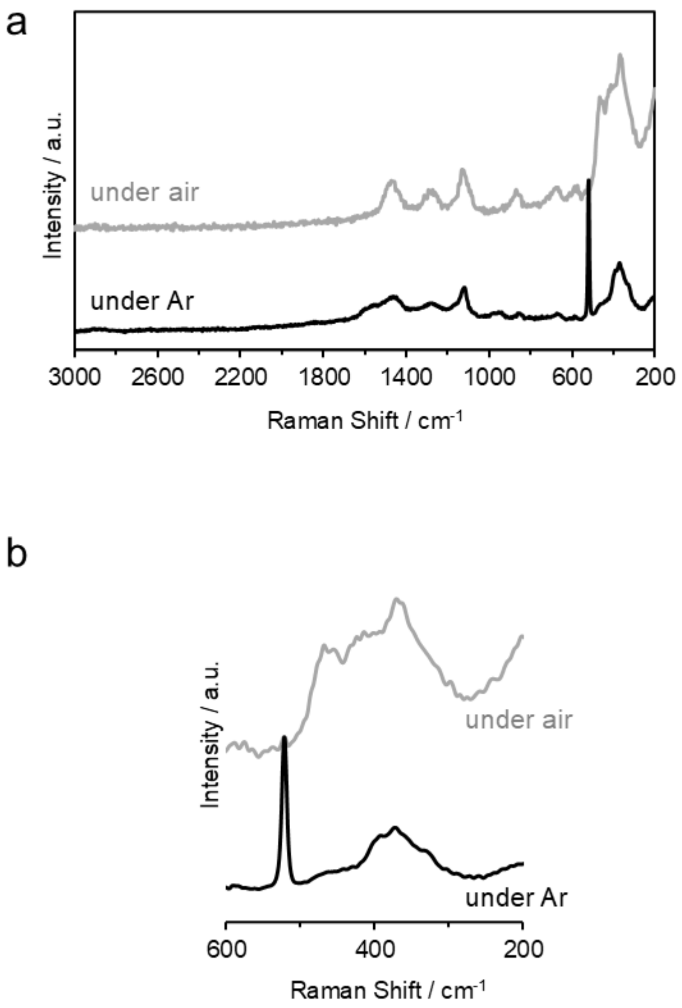

Figure S6. Raman spectra of NiDT without unreacted BHT. a, 200-3000 cm^{-1}. **b,** 200-600 cm^{-1}. The intense peak at 521 cm^{-1} is ascribed to Si.

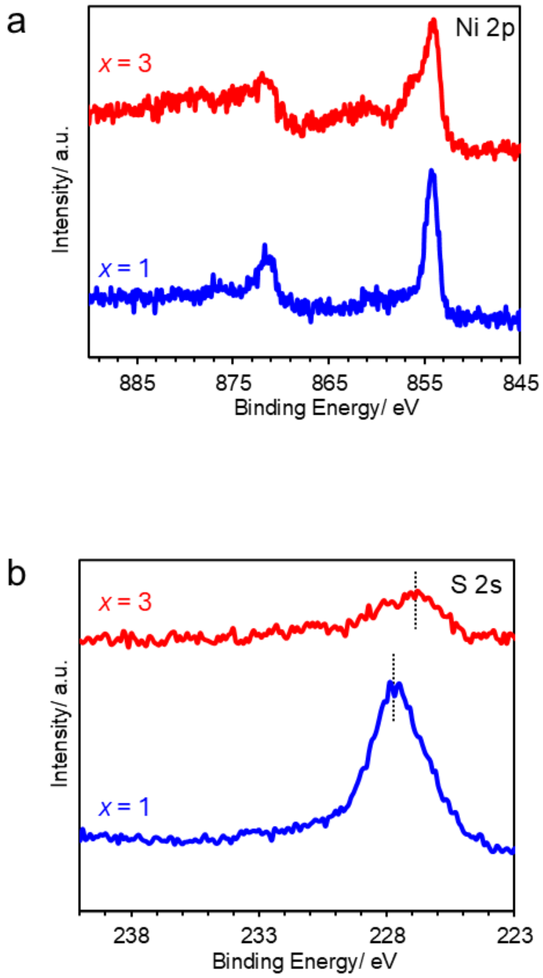

Figure S7. **XP spectra of Ni_1/BHT and Ni_3/BHT. a,** Ni 2p. **b,** S 2s.

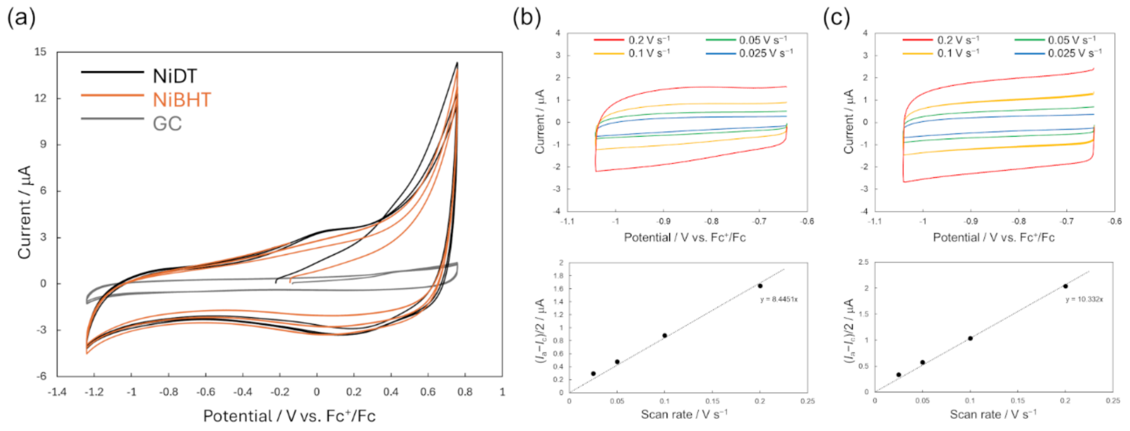

Figure S8. **Electrochemical measurements of NiDT and NiBHT. a,** Cyclic voltammograms of NiDT (black line), NiBHT (orange line), and glassy carbon (gray line) recorded at a scan rate of 0.1 V s^{-1}. Cyclic voltammograms recorded at various scan rates and the calculation of electric double layer capacitances of NiDT (**b**) and NiBHT (**c**). Anodic (I_a) and cathodic currents (I_c) at −0.842 V vs. Fc^+/Fc were used for the capacitance calculation.

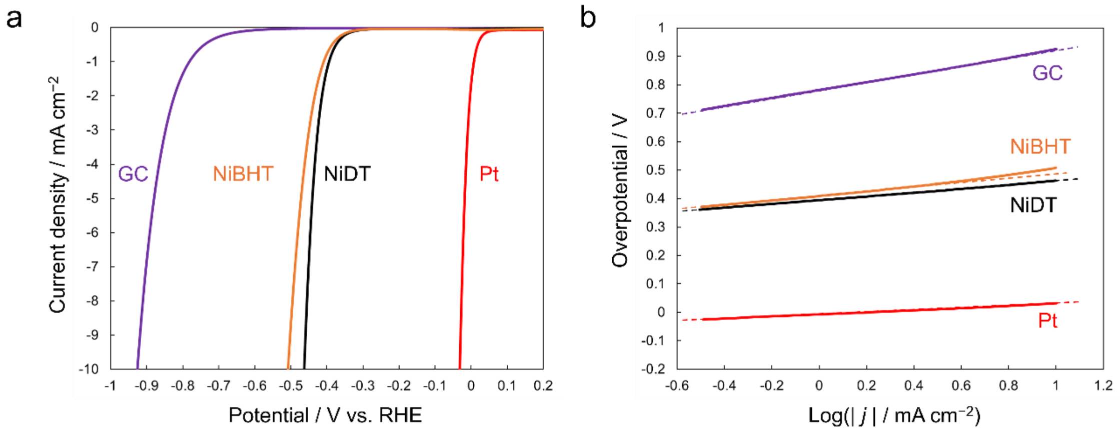

c

Entry	E_{onset} / mV	$\eta_{j=10}$ / mV	Tafel slope
Pt	7	32	37
NiDT	−395 ± 3	467 ± 6	70 ± 8
NiBHT	−405 ± 16	505 ± 10	86 ± 13
GC	−782	803	141

Figure S9. **Electrocatalytic performance of NiDT and NiBHT. a,** linear sweep voltammetry of NiDT, NiBHT, bare glassy carbon, and platinum. **b,** their Tafel plot. **c,** the summary of the onset potential, overpotential, and Tafel slope.

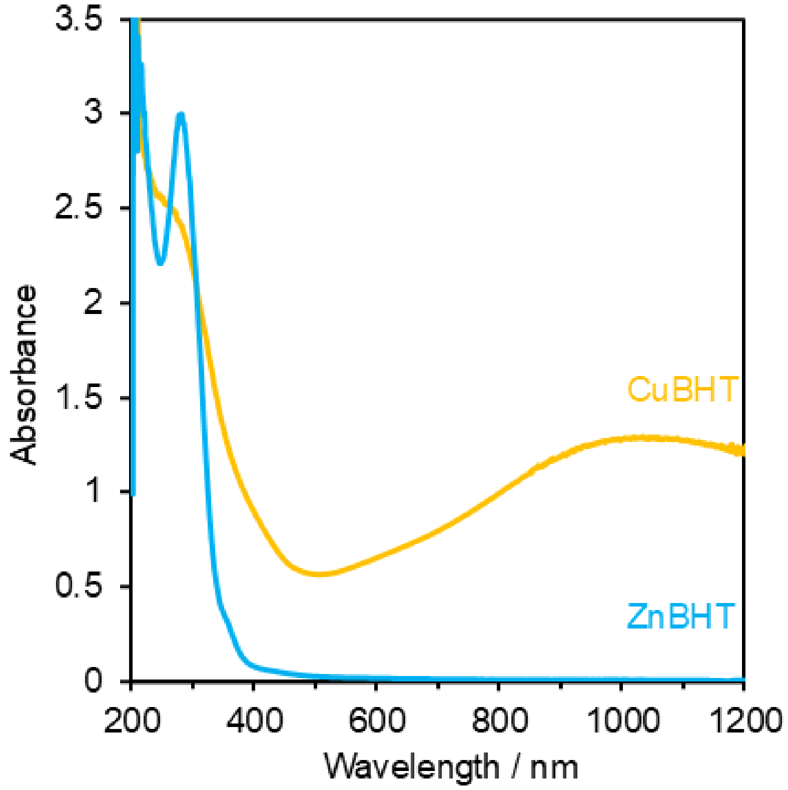

Figure S10. UV–Vis–NIR absorption spectra of MBHT (M= Cu, Zn).

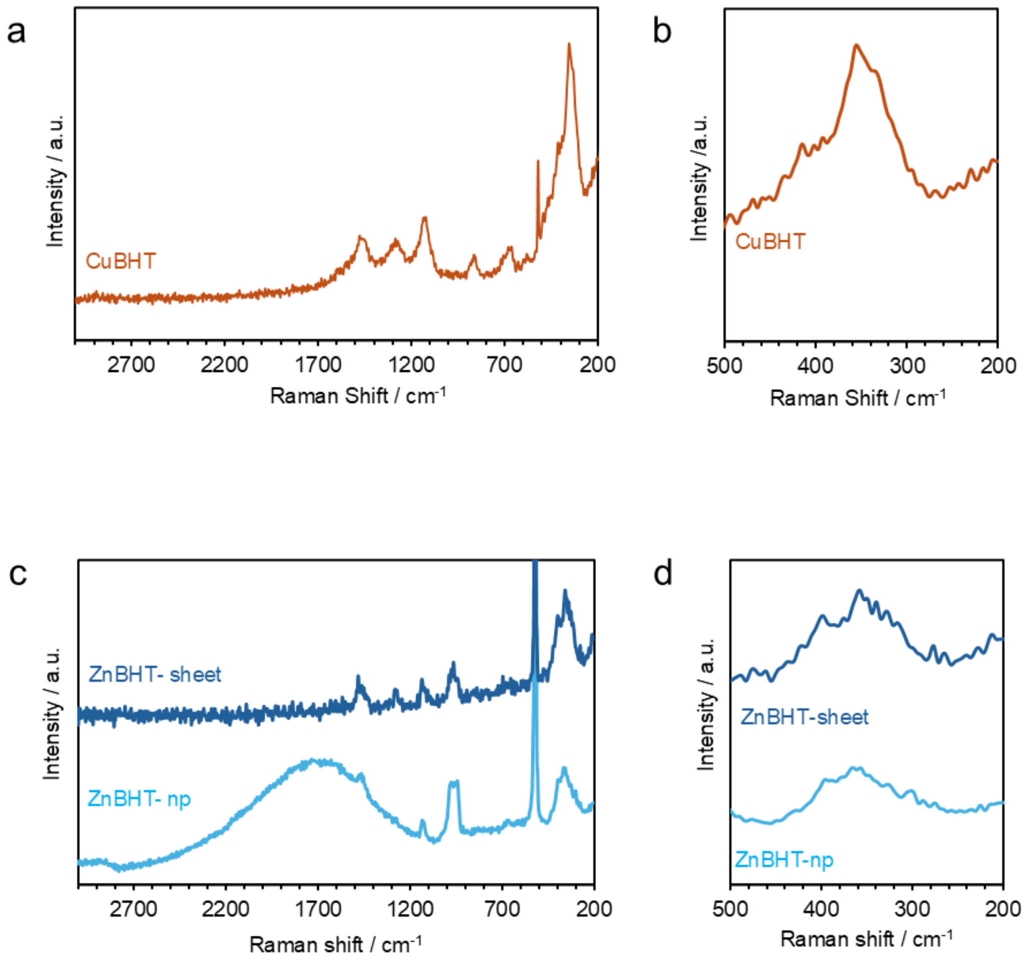

Figure S11. Raman spectra of MBHT. a, CuBHT in 200-3000 cm^{-1}. **b,** CuBHT in 200-500 cm^{-1}. **c,** ZnBHT of a single-phase synthesis method (np) and liquid-liquid interfacial synthesis (sheet) in 200-3000 cm^{-1}. **d,** 200-500 cm^{-1}. The intense peak at 521 cm^{-1} is ascribed to Si.

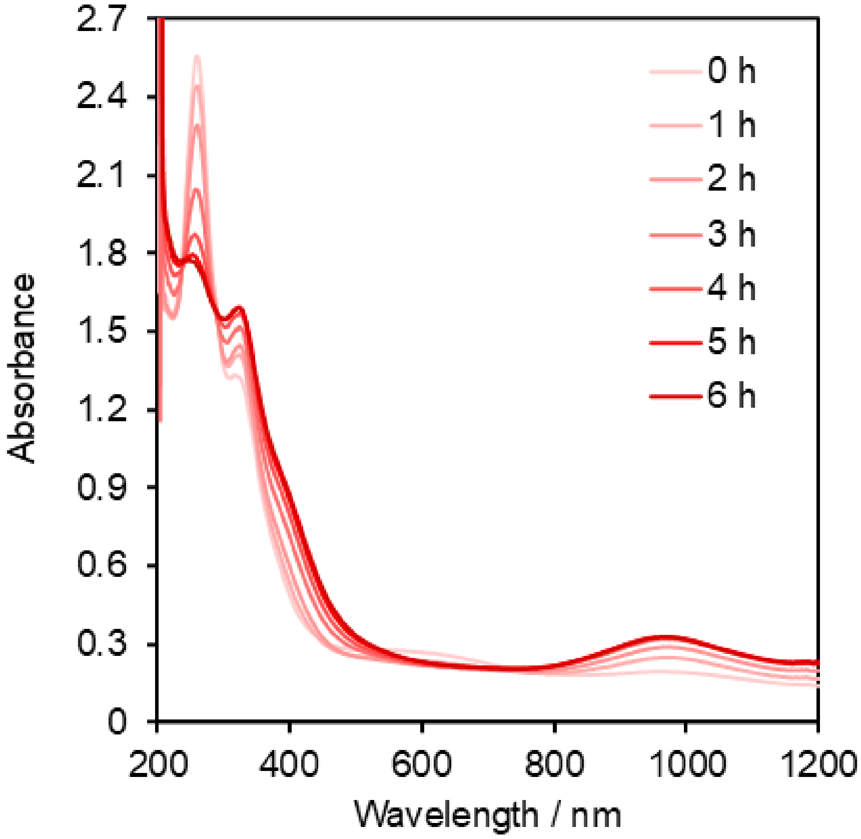

Figure S12. Time-dependent UV-Vis-NIR absorption spectra of Ni_1/BHT from 0 to 6 h.

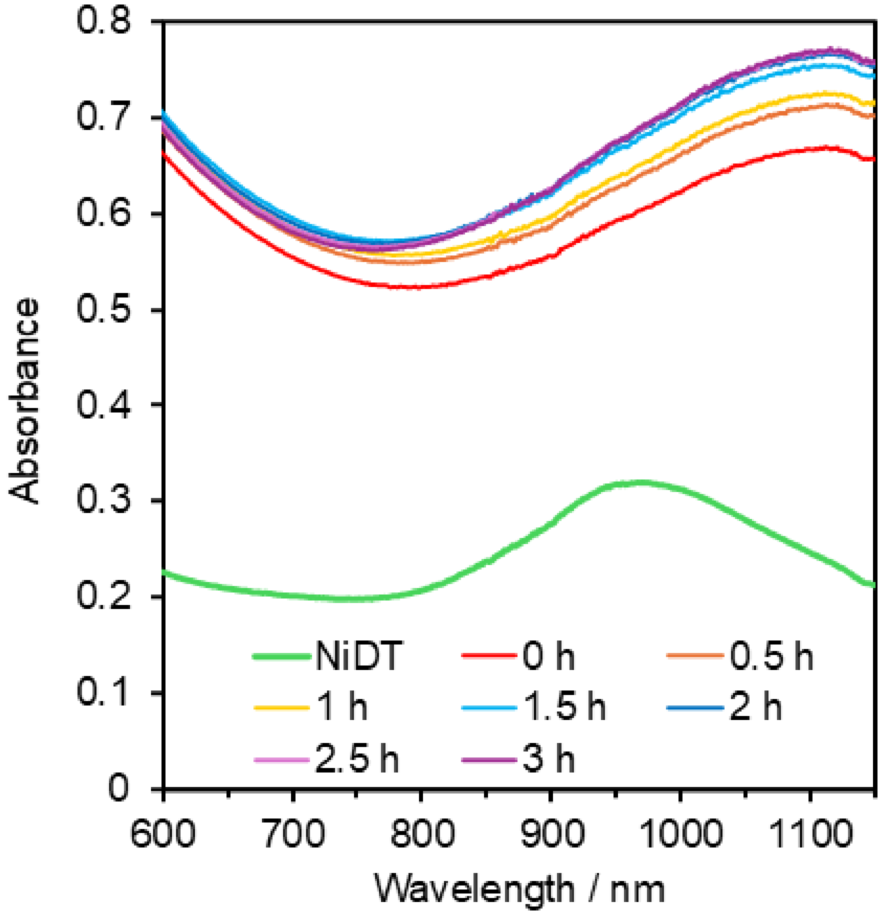

Figure S13. Vis-NIR absorption spectra during the reaction from NiDT to NiBHT.

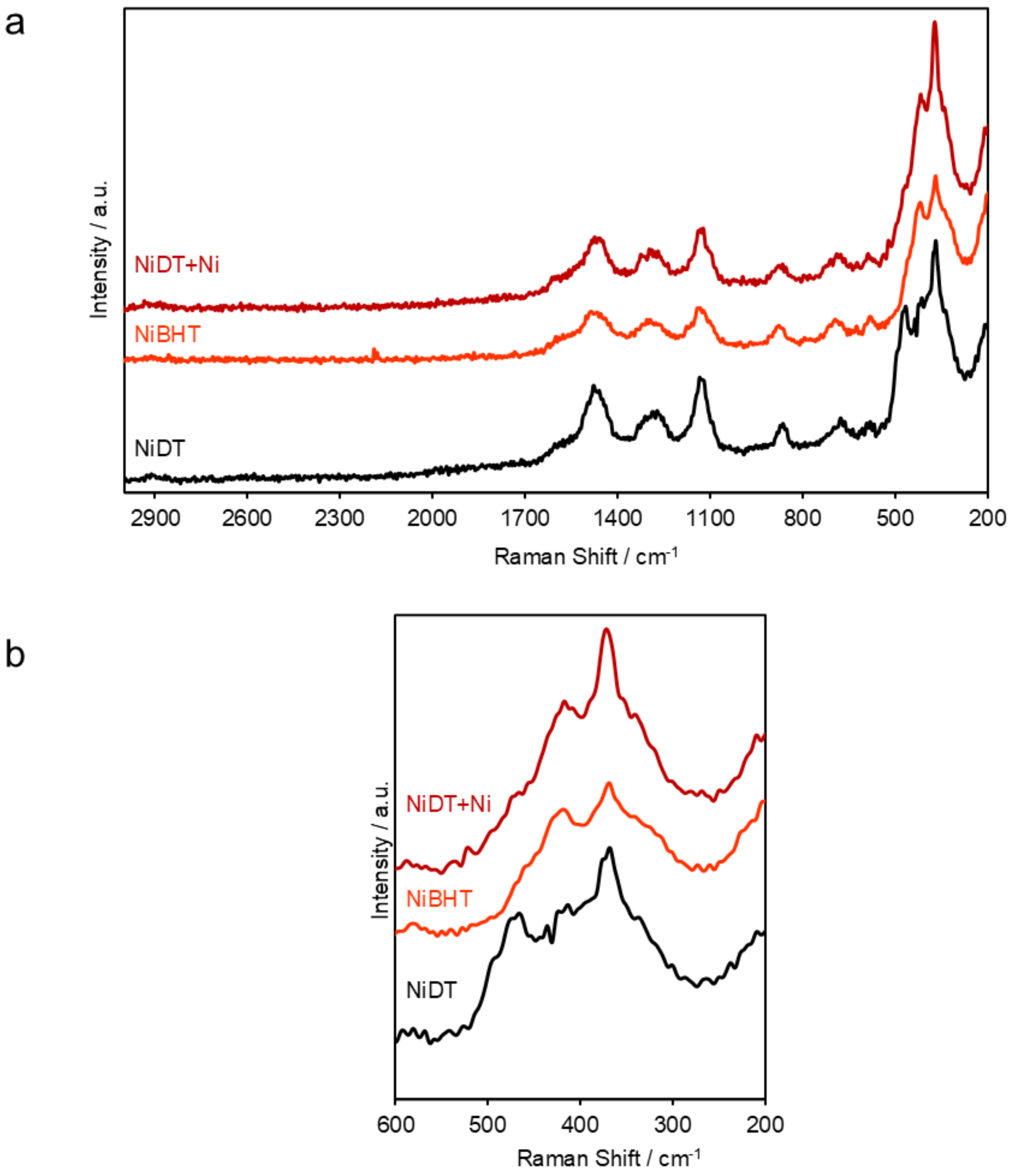

Figure S14. Raman spectra of NiDT, NiBHT, and NiDT+Ni. a, 200-3000 cm^{-1}. **b,** 200-600 cm^{-1}.

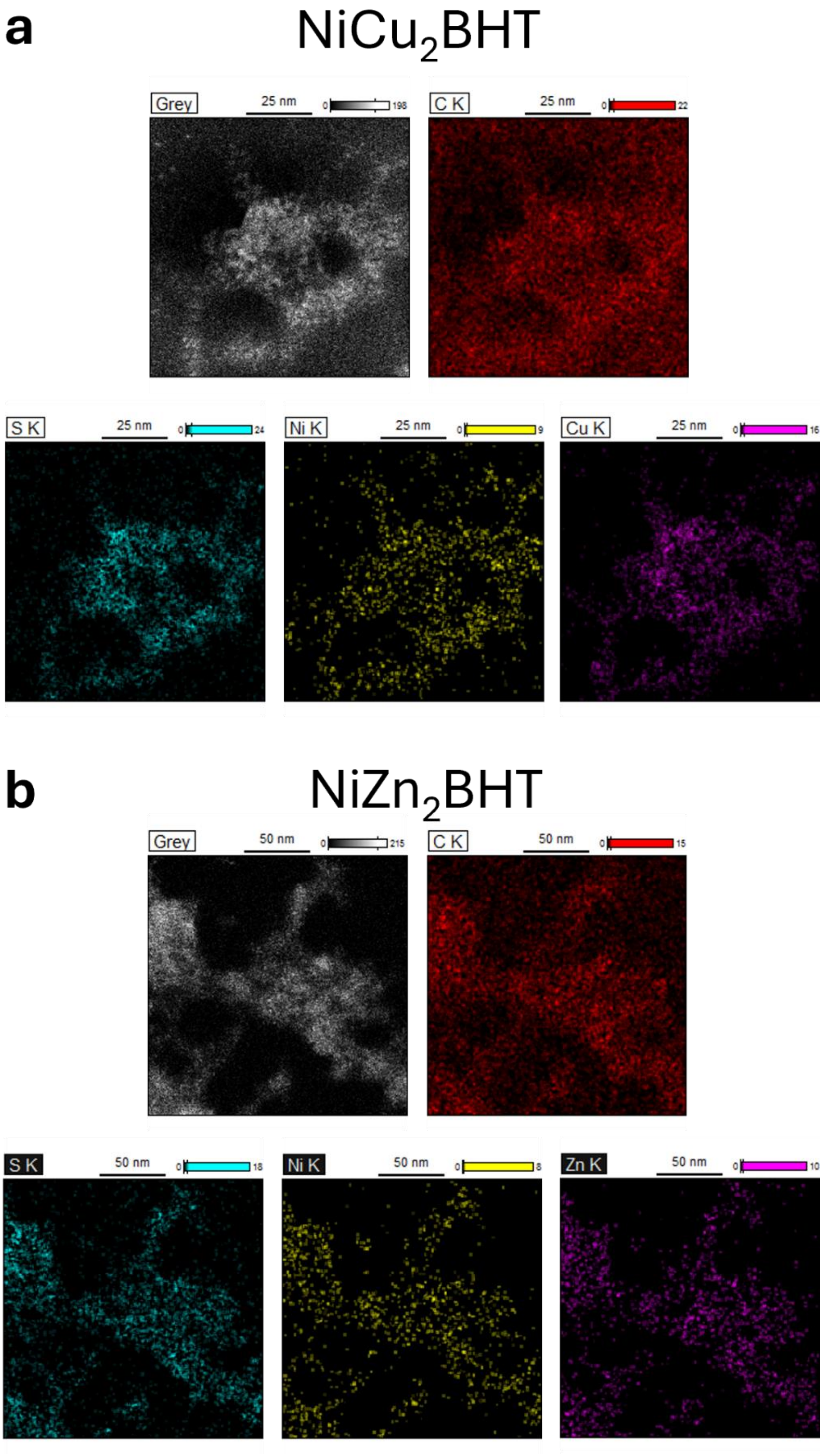

Figure S15. STEM-EDS of NiM_2BHT. a, $NiCu_2BHT$. **b,** $NiZn_2BHT$.

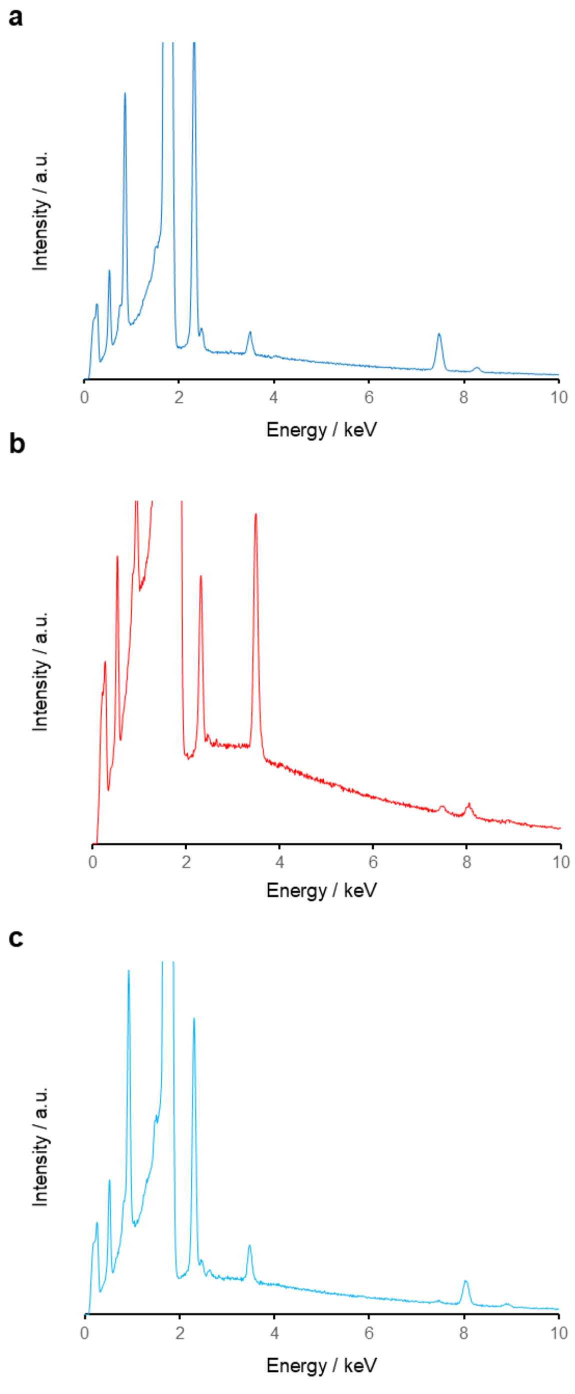

Figure S16. SEM-EDS of MBHT nanofilms. a, NiBHT. **b,** tm-$NiCu_2BHT$. **c,** tm-CuBHT.

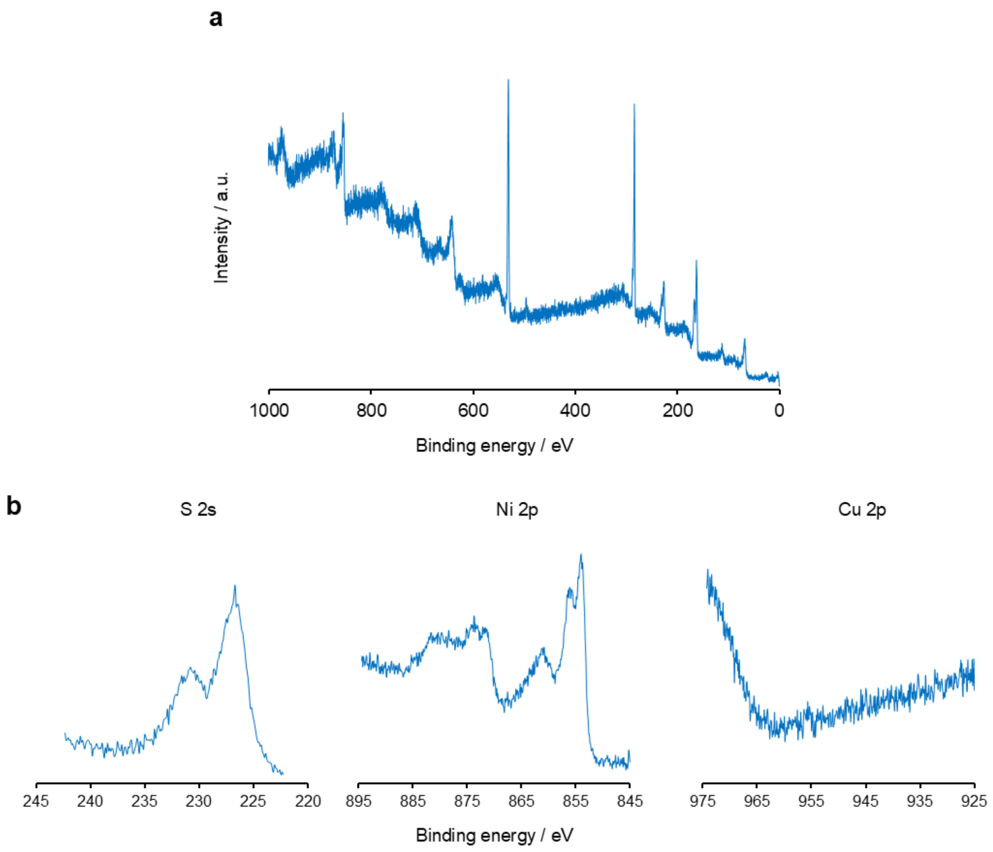

Figure S17. XP spectrum of NiBHT nanofilm. a, survey scan. **b,** narrow scan.

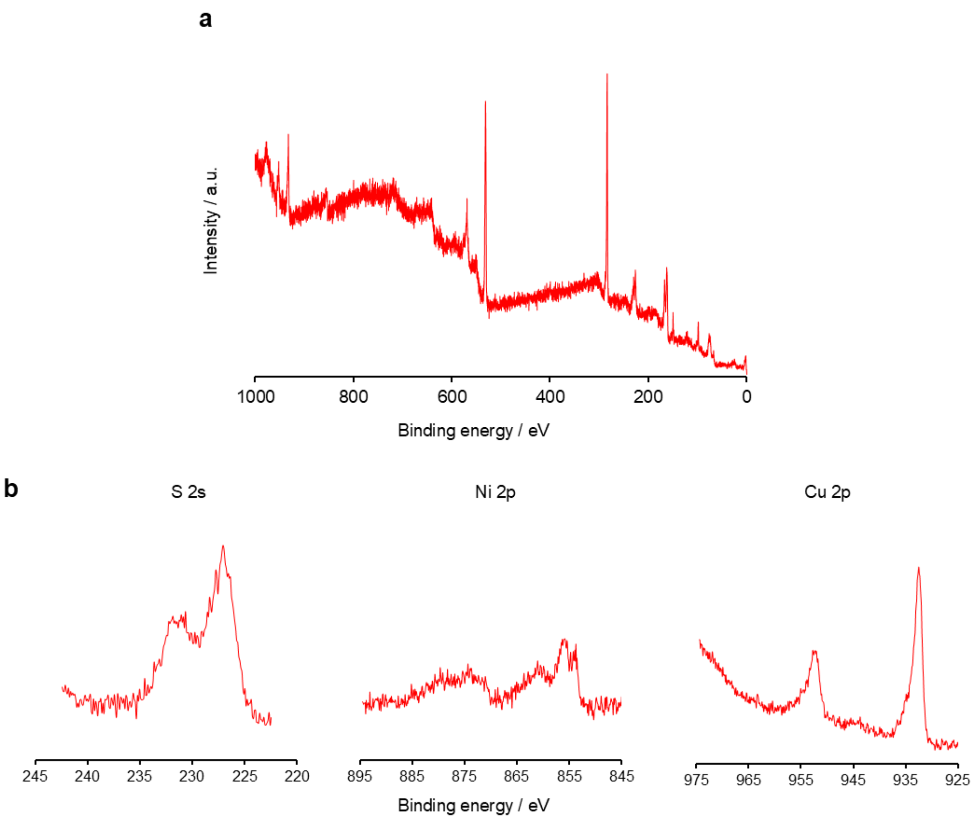

Figure S18. XP spectrum of tm-$NiCu_2$BHT. a, survey scan. **b,** narrow scan.

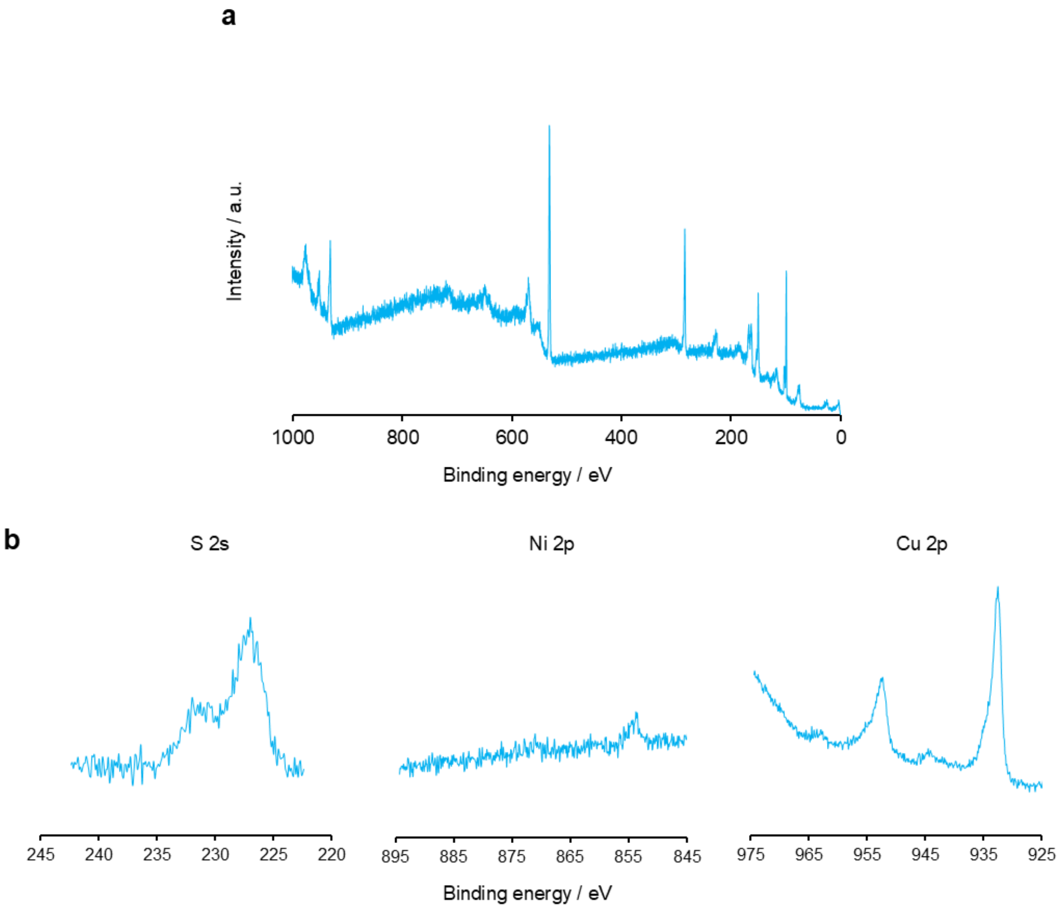

Figure S19. XP spectrum of tm-CuBHT. a, survey scan. **b,** narrow scan.

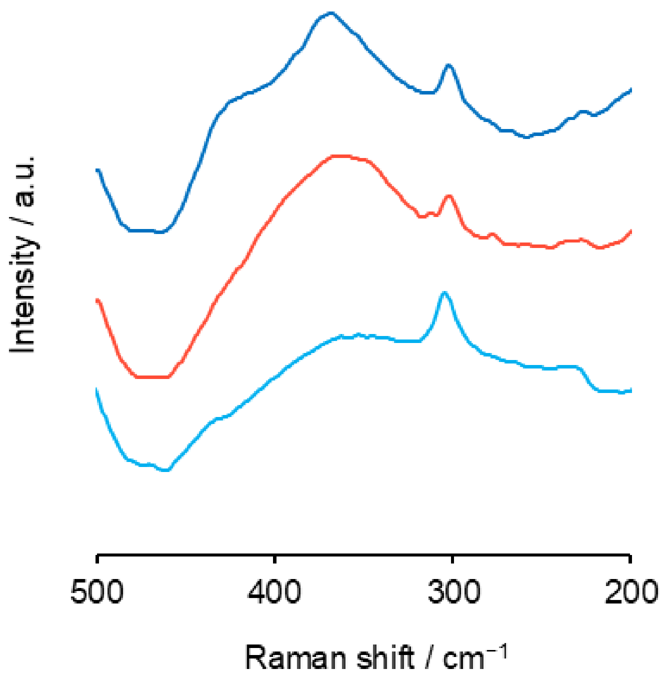

Figure S20. Raman spectra of MBHT nanofilms. NiBHT (Blue), tm-$NiCu_2BHT$ (red), and tm-CuBHT (light blue).

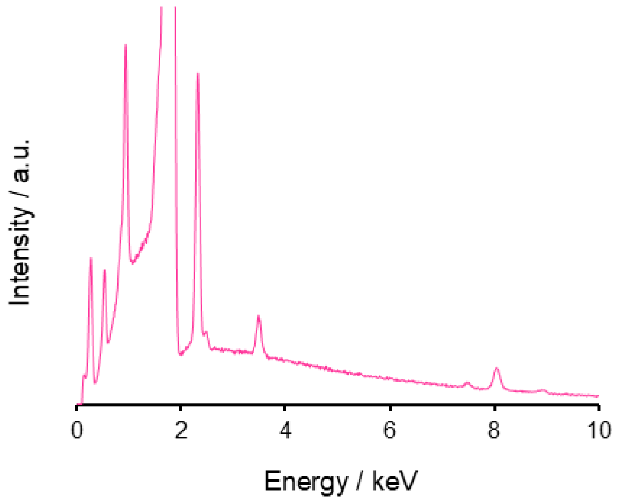

Figure S21. SEM-EDS of transmetallated CuBHT nanofilm with $NiCl_2$.

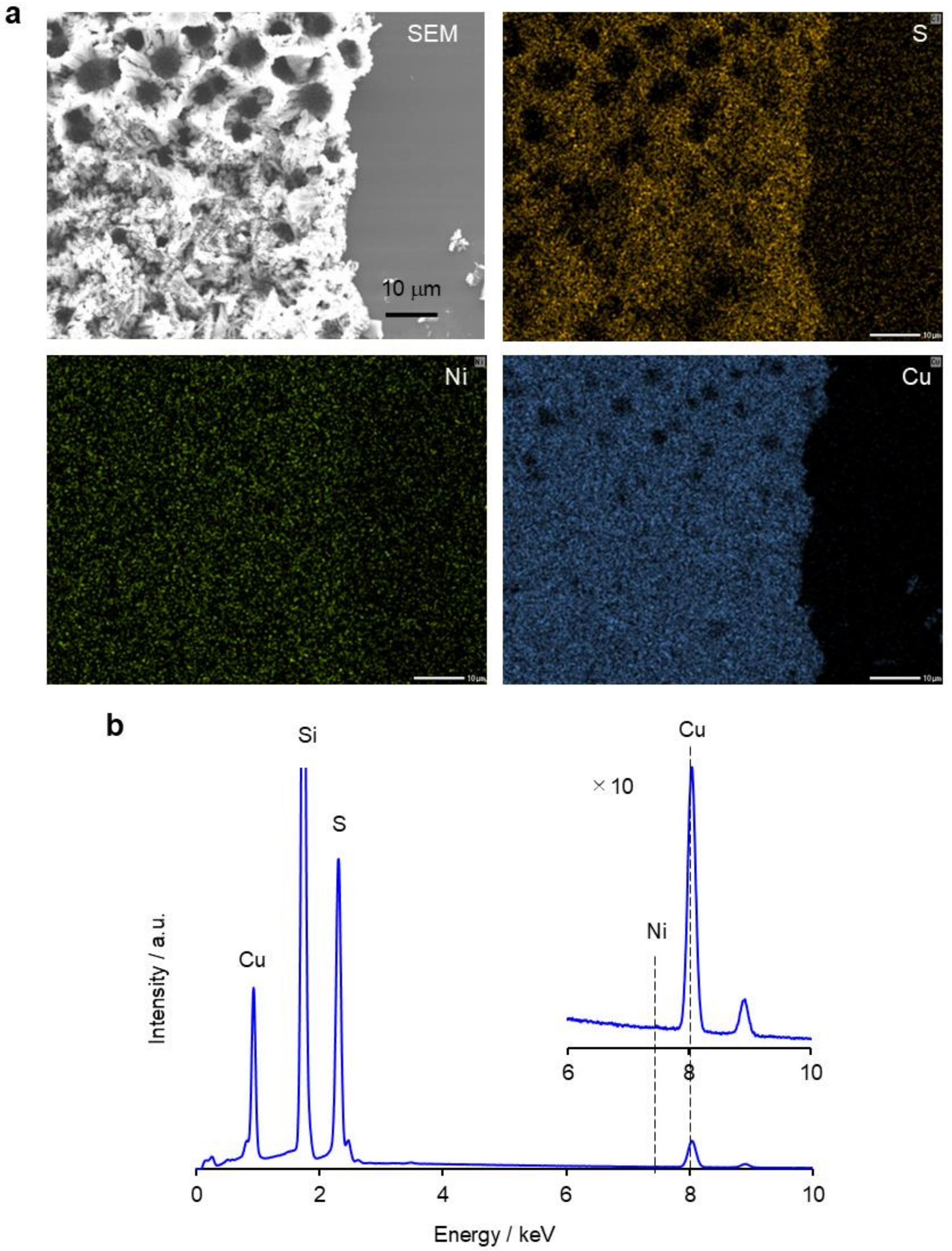

Figure S22. SEM-EDS of $NiCu_2BHT$ prepared at 80 °C. a, SEM-EDS elemental mappings. **b,** SEM-EDS spectrum.

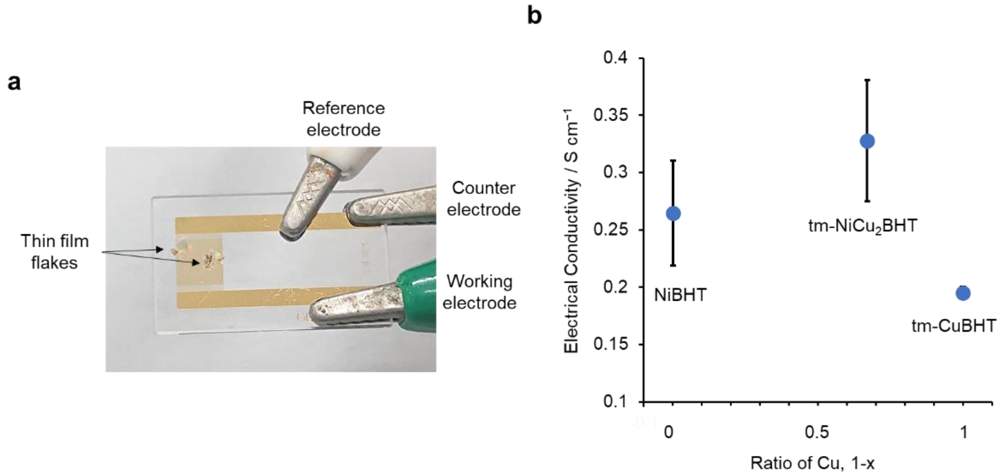

Figure S23. Relations between electrical conductivity and ratio of transmetallated Cu^{2+} ions. a, Experimental setup for electrical conductivity measurements. **b,** electrical conductivities of NiBHT, tm-$NiCu_2BHT$, and tm-CuBHT thin films.
